# An enriched environment ameliorates the reduction of parvalbumin-positive interneurons in the medial prefrontal cortex caused by maternal separation early in life

**DOI:** 10.3389/fnins.2023.1308368

**Published:** 2024-01-16

**Authors:** Kanako Irie, Ken-ichi Ohta, Hidetoshi Ujihara, Chihiro Araki, Kodai Honda, Shingo Suzuki, Katsuhiko Warita, Hikari Otabi, Haruki Kumei, Shinji Nakamura, Kosuke Koyano, Takanori Miki, Takashi Kusaka

**Affiliations:** ^1^Department of Anatomy and Neurobiology, Faculty of Medicine, Kagawa University, Kagawa, Japan; ^2^Department of Pediatrics, Faculty of Medicine, Kagawa University, Kagawa, Japan; ^3^Department of Veterinary Anatomy, Faculty of Agriculture, Tottori University, Tottori, Japan

**Keywords:** maternal separation, medial prefrontal cortex, sensory cortex, parvalbumin, enriched environment, critical period, perineuronal net

## Abstract

Early child maltreatment, such as child abuse and neglect, is well known to affect the development of social skills. However, the mechanisms by which such an adverse environment interrupts the development of social skills remain unelucidated. Identifying the period and brain regions that are susceptible to adverse environments can lead to appropriate developmental care later in life. We recently reported an excitatory/inhibitory imbalance and low activity during social behavior in the medial prefrontal cortex (mPFC) of the maternal separation (MS) animal model of early life neglect after maturation. Based on these results, in the present study, we investigated how MS disturbs factors related to excitatory and inhibitory neurons in the mPFC until the critical period of mPFC development. Additionally, we evaluated whether the effects of MS could be recovered in an enriched environment after MS exposure. Rat pups were separated from their dams on postnatal days (PDs) 2–20 (twice daily, 3 h each) and compared with the mother-reared control (MRC) group. Gene expression analysis revealed that various factors related to excitatory and inhibitory neurons were transiently disturbed in the mPFC during MS. A similar tendency was found in the sensory cortex; however, decreased parvalbumin (PV) expression persisted until PD 35 only in the mPFC. Moreover, the number of PV^+^ interneurons decreased in the ventromedial prefrontal cortex (vmPFC) on PD 35 in the MS group. Additionally, perineural net formation surrounding PV^+^ interneurons, which is an indicator of maturity and critical period closure, was unchanged, indicating that the decreased PV^+^ interneurons were not simply attributable to developmental delay. This reduction of PV^+^ interneurons improved to the level observed in the MRC group by the enriched environment from PD 21 after the MS period. These results suggest that an early adverse environment disturbs the development of the mPFC but that these abnormalities allow room for recovery depending on the subsequent environment. Considering that PV^+^ interneurons in the mPFC play an important role in social skills such as empathy, an early rearing environment is likely a very important factor in the subsequent acquisition of social skills.

## 1 Introduction

A nurturing environment early in life is an important factor in the development of social skills, and early adverse environments, such as child abuse and neglect, cause poor sociability or peer interaction (Darwish et al., 2001; Lum et al., 2018; Zhang et al., 2023). Moreover, emotional neglect attenuates empathy (Berzenski and Yates, 2022; Chen et al., 2022) and recognition of facial expressions (Pollak et al., 2000) more than physical abuse. Similarly, some studies using the animal maternal separation (MS) model, which is commonly used as an animal model of early life neglect, have reported that MS during early brain development attenuates social behavior (Ognibene et al., 2008; Franklin et al., 2011; Yang et al., 2017). We also reported that maternal separation (MS) attenuates social behavior related to social recognition, which is important for social skills after maturation (Ohta et al., 2020).

Such social dysfunction is caused by abnormalities in various brain regions, including the medial prefrontal cortex (mPFC). The mPFC is strongly associated with social cognition, such as empathy for others and the perception of self and others (Mitchell et al., 2006; Shamay-Tsoory et al., 2009; Bicks et al., 2015; Beadle et al., 2018). In addition, rodent studies have indicated that the mPFC is closely related to social behavior, particularly social recognition, to distinguish novel from familiar conspecifics (Lee et al., 2016; Tan et al., 2019). In human neuroimaging studies, child maltreatment causes structural and neural activation abnormalities in several brain regions, among which the mPFC is one of the brain areas vulnerable to an early adverse environment. Specifically, youths who have previously experienced maltreatment exhibit a smaller mPFC volume (Gold et al., 2016; Sheridan et al., 2022) or less activity during emotional tasks (Jenness et al., 2021). Additionally, we recently reported that the prolonged MS model showed attenuated neural activity in the mPFC during the social behavior test (Ohta et al., 2020). We also demonstrated that prolonged MS causes an imbalance in excitatory/inhibitory neurons and decreases parvalbumin (PV)^+^ interneurons in the mPFC after maturation (Ohta et al., 2020). PV^+^ interneurons, classified as fast-spiking gamma-aminobutyric acid (GABA) ergic interneurons, play a role in synchronizing cortical neurons. The synchronized oscillations formed by PV^+^ interneurons (gamma oscillations) in the mPFC play crucial roles in social recognition (Cao et al., 2018). Considering these studies, the mPFC could be a crucial aspect for clarifying the mechanisms of subsequent social dysfunction in early adverse environments.

In rodents, whole-brain weight rapidly increases during the first two weeks, which is equivalent to the time from the third trimester of pregnancy to birth in humans (Dobbing and Sands, 1979; Bethlehem et al., 2022). This period is called the brain growth spurt, in which the brain reaches 90% of the adult weight by around postnatal day (PD) 21 in rodents (equivalent to approximately 2–3 years of age in humans) (Semple et al., 2013; Rodriguez-Gonzalez et al., 2020). The gray matter volume in the whole brain, neocortex (mainly including the motor) and sensory cortex (SC), hippocampus, and cerebellum also reaches the adult level at around PD 20 in rodents (Chuang et al., 2011), whereas that of the cerebrum in humans reaches the adult level in early childhood, around 2–3 years of age (Bethlehem et al., 2022). However, volumetric development in the mPFC peaks later than that in other brain regions, at around PD 24 (Van Eden and Uylings, 1985). Similarly, in human studies, gray matter volume and synaptic density in the frontal cortex peak several years after those in the whole brain or other brain regions, such as the visual cortex, SC, and auditory cortex (Huttenlocher and Dabholkar, 1997; Matsuzawa et al., 2001; Bethlehem et al., 2022). Moreover, the critical period in the mPFC, during which higher neural plasticity is maintained and the neural circuit is formed and refined, is later than in other brain regions, such as the SC and amygdala (Lendvai et al., 2000; Gogolla et al., 2009; Lo et al., 2017; Bicks et al., 2020; Reh et al., 2020). The formation of the perineural net (PNN) surrounding PV^+^ interneurons, an indicator of the maturation of PV^+^ interneurons and critical period closure (Reichelt et al., 2019), reaches the adult level on PD 35 in the mPFC and later than on PD 24 in the amygdala (Baker et al., 2017). Therefore, the influences of an early adverse environment are likely to allow room for recovery in the mPFC until preadolescence, compared to other brain regions. However, when and how early adverse environments interrupt the development of the mPFC and whether their influences on the mPFC are permanent remain poorly understood.

Based on our previous study (Ohta et al., 2020), we investigated how MS during early brain development affects the developmental trajectory of excitatory and inhibitory neurons in the mPFC until PD 35, which corresponds to the critical period of closure of the mPFC. In addition, we investigated whether the influence of MS on the mPFC during early brain development could be improved by changing the environment after MS. For comparison, we analyzed the SC, whose critical period closes during the MS period in this study, unlike the mPFC, and examined the differences in impact between the SC and the mPFC. MS transiently interrupted the gene expression of various factors related to excitatory and inhibitory neurons in both the SC and mPFC, with similar trends observed during the first and second postnatal weeks. However, PV gene expression in the mPFC was persistently reduced until PD 35 despite the termination of MS on PD 20. Moreover, PV^+^ interneurons were decreased only in the ventral part of the mPFC on PD 35. This reduction was reversible and could be recovered by an enriched environment (EE) after MS.

## 2 Materials and methods

### 2.1 Animals

**Figure 1:**
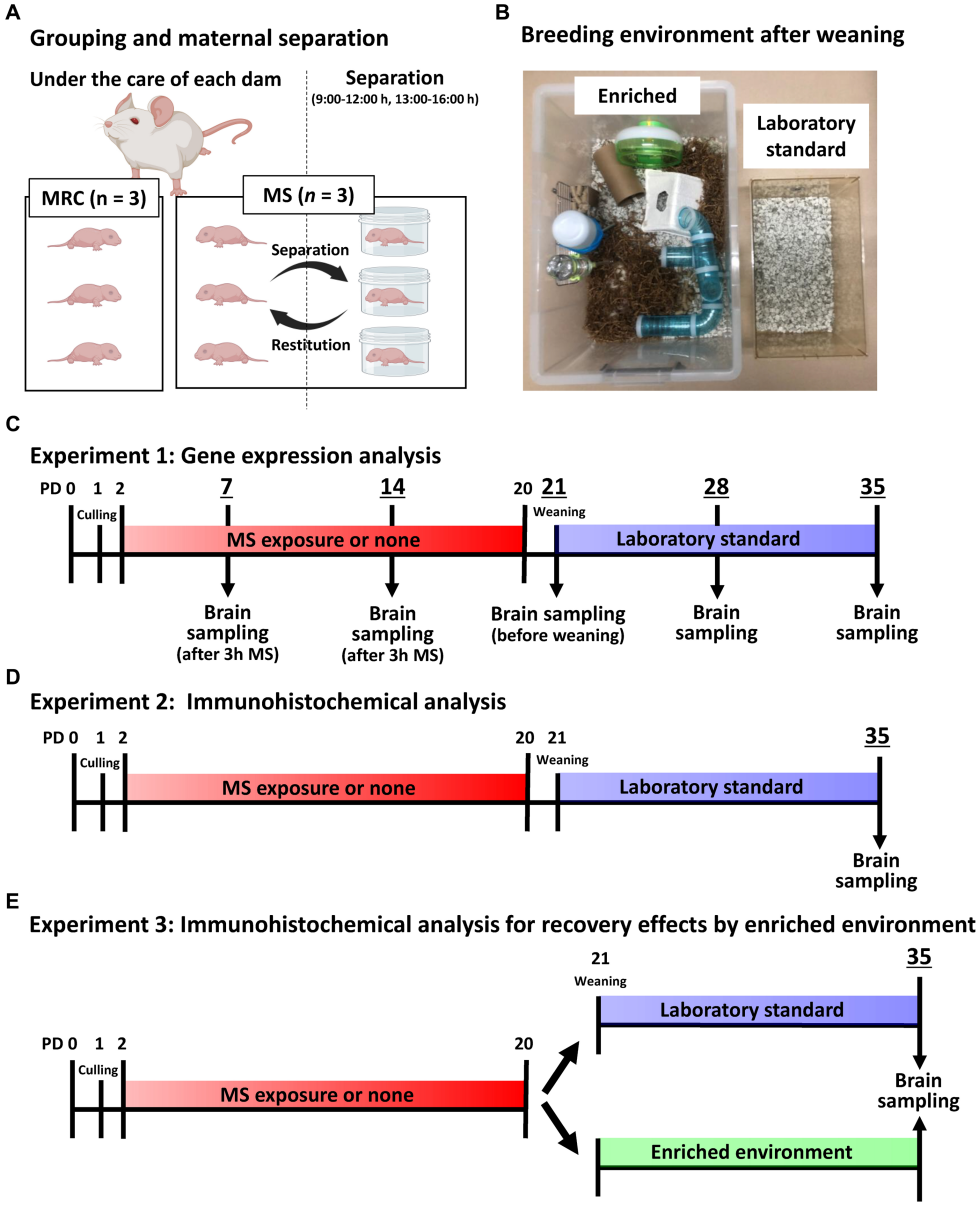
Image and timeline of the experimental design. **(A)** An illustration of the grouping per dam and maternal separation (MS). MRC: mother-reared control. **(B)** Image showing the enriched and laboratory standard environments after weaning. **(C)** Timeline of gene expression analysis until brain sampling on each postnatal day (PD). **(D)** Timeline of immunohistochemical analysis on PD 35 until brain sampling. **(E)** Timeline of MS and enriched environment exposure for immunohistochemical analysis on PD 35 until brain sampling.

All experiments were approved by the Animal Care and Use Committee of Kagawa University (approval number:21633). Sprague–Dawley rats (RRID: RGD_12910483) were used for all analyses, and pregnant rats were purchased from Japan SLC Inc. (Hamamatsu, Japan). These rats were individually housed in plastic cages in a temperature-controlled room (22 ± 2°C) with a 12-h light/dark cycle (lights on from 06:00 to 18:00 h) and had access to food and water *ad libitum*. Pups were obtained through spontaneous delivery. Gestational day (GD) 0 was set as the day the plug was confirmed. The pregnant rats were checked not to give birth at 17:00 h on GD 21, and then, when were checked again at 09:00 h on GD 22, the pups that had already been born were designated as PD 0. On PD 1, two pups from another dam were distributed to the dam from which all pups were removed so that each dam had eight pups (male/female = 6/2). Thus, each dam fostered pups from three different litters. On PD 2, one of the two male pups collected from each dam was assigned to the MS group and the other to the mother-reared control (MRC) group. Therefore, each dam simultaneously fostered the MS (three pups) and MRC (three pups) groups (Figure 1A). Randomization was not employed in this study; however, efforts were made to ensure the uniform body weight of pups before MS, dam care, and genetic background between the groups.

### 2.2 MS procedure and breeding in an enriched environment

The MS procedure was based on previous studies (Ohta et al., 2023). Specifically, the rats in the MS group were individually isolated in plastic cases at 22 ± 2°C for 3 h twice daily (from 09:00 to 12:00 h and from 13:00 to 16:00 h) between PD 2 and 20 (Figure 1A). They were returned to their dams between 12:00 and 13:00 h to avoid negatively affecting their nutritional status. We previously confirmed that the MS group did not experience malnutrition, and the serum corticosterone level of the MS group increased fourfold during the separation period compared with that of the MRC group (Ohta et al., 2014). The rats in the MRC group remained in their home cages with the dams and were not handled, except for changing their cage bedding on PD 4, 8, and 15.

To eliminate possible influences of the sexual cycle, male offspring were used for all analyses. All male offspring were weaned on PD 21, and two male offspring from the same-rearing group were housed in plastic cages (CL-108-3: 276 × 445 × 204 mm; CLEA Japan, Tokyo, Japan) with standard bedding (Eco chip: CL-4163; CLEA Japan, Tokyo, Japan) as laboratory standard. In the EE experiment, a group of six male offspring from the same-rearing group were housed in a large cage (440 × 660 × 320 mm) with nesting material (Enviro-dri; Shepherd Specialty Papers, New Jersey, United States), nest box (Shepherd Shack and Shepherd tube; Shepherd Specialty Papers), and toys, such as running wheel and tubular maze, until PD 35 (Figure 1B).

### 2.3 Gene expression analysis

Tissues were sampled as previously described (Ohta et al., 2014, 2017). After measuring the body weight, the pups from both groups were anesthetized with isoflurane (Cat. No. 099–06571; FUJIFILM Wako Pure Chemical, Osaka, Japan) and intracardially perfused with medical-grade physiological saline on PDs 7, 14, 21, 28, and 35 (Figure 1C). Sampling of the MS group on PD 7 and 14 was performed immediately after 3 h of separation to accurately estimate the changes during MS. All samples were obtained between 12:00 and 14:00 to minimize the effect of circadian change. After perfusion, the brains were removed from the skull. The brain weights were measured, and the brains were sectioned in the coronal plane to yield 1-mm-thick slices using a Brain Matrix device (Roboz Surgical Instrument, Maryland, United States). Bilateral SC and mPFC were dissected from each slice using a stereoscopic microscope (Leica Geosystems, Heerbrugg, Switzerland). The SC was collected as an enriched sample using an indicator at the top of the hippocampus and the bottom of the fimbria. The mPFC was collected using indicators at the top and bottom of the forceps minor of the corpus callosum. The samples were stored at −80°C until required.

Tissue homogenization and total RNA extraction were performed using the RNeasy Mini Kit (Qiagen, Hilden, Germany) according to the manufacturer’s protocol. The concentration and purity of the extracted total RNA were evaluated by measuring the optical density at 260 and 280 nm using a NanoDrop 1,000 (Thermo Fisher Scientific, Waltham, MA, United States). A QuantiTect Reverse Transcription Kit (Qiagen) was used to synthesize cDNA with integrated genomic DNA removed from 500 ng of the total RNA sample. Gene expression was quantified using a ViiA™7 system (Thermo Fisher Scientific) with Fast SYBR Green Master Mix (Thermo Fisher Scientific). To assess differences in gene expression during development, samples from PD 7 to PD 35 were measured simultaneously in the same 96-well polymerase chain reaction (PCR) plate (Watson, Hyogo, Japan). The primer pairs used in this study are listed in Table 1. The primer pairs, except for calcium/calmodulin-dependent protein kinase type II subunit alpha (CaMKIIα) and PV, were as reported in our previous studies (Ohta et al., 2017, 2020). The amount of each mRNA was estimated by normalization to glyceraldehyde 3-phosphate dehydrogenase (GAPDH) mRNA levels in the same sample.

**Table 1:** Forward and reverse primers used for real-time RT-PCR.

Target gene		Primer sequence	Product size (bp)	Accession No.
*CaMKIIα*				
	Forward	5′-ATATCGTCCGACTCCATGAC-3′	98	NM_012920.1
	Reverse	5′-GGCCACAATGTCTTCGAAC-3′		
*GAD65*				
	Forward	5′-CTGCTTCTGGTTTGTACCTCCT-3′	122	NM_012563.1
	Reverse	5′-CCATTGTGGTCCCATACTCC-3′		
*GAD67*				
	Forward	5′-CTGGAGCTGGCTGAATACCT-3′	120	NM_017007.1
	Reverse	5′-TCGGAGGCTTTGTGGTATGT-3′		
*GAPDH*				
	Forward	5′-ATGGCCTTCCGTGTTCCTAC-3′	53	NM_017008.4
	Reverse	5′-CGGCATGTCAGATCCACAAC-3′		
*Gephyrin*				
	Forward	5′-AAAGATGGCTATGCTGTTCGAG-3′	99	NM_022865.3
	Reverse	5′-GGGCATTACTGTCTGAGTTGG-3′		
*NLGN1*				
	Forward	5′-TTGGCTGCAATGTGTCAG-3′	114	NM_053868.2
	Reverse	5′-GTCCAAAGGCTATGTGGTATC-3′		
*NLGN2*				
	Forward	5′-CCAAAGTGGGCTGTGACC-3′	118	NM_053992.1
	Reverse	5′-CCAAAGGCAATGTGGTAGC-3′		
*NR2A*				
	Forward	5′-GACGGTCTTGGGATCTTAAC-3′	139	NM_012573.3
	Reverse	5′-TGACCATGAATTGGTGCAGG-3′		
*NR2B*				
	Forward	5′-TTTGGCCCGTCTATCGAAC-3′	146	NM_012574.1
	Reverse	5′-AAGCTGTTCTCGATGGTACTG-3′		
*PSD95*				
	Forward	5′-GCAGGTTGCAGATTGGAGAC-3′	121	NM_019621.1
	Reverse	5′-GCCACCTTTAGGTACACAACG-3′		
*PV*				
	Forward	5′-ACATCAAGAAGGCGATAGGAG-3′	120	M12725.1
	Reverse	5′-GAATGTGGAACACCTTCTTCAC-3′		
*VGAT*				
	Forward	5′-GGGCTGGAACGTGACAAA-3′	65	AF030253.1
	Reverse	5′-GGAGGATGGCGTAGGGTAG-3′		
*VGLUT1*				
	Forward	5′-GTCATGACTATCATCGTACCCATC-3′	122	NM_053859
	Reverse	5′-GTAGCTTCCATCCCGAAACC-3′		
*VGLUT2*				
	Forward	5′-GCAAGGTTGGCATGTTGTC-3′	94	NM_053427.1
	Reverse	5′-TGCTTGCTCCTTAGAAAGTCTG-3′		

CaMKIIα, calcium/calmodulin-dependent protein kinase type II subunit alpha; GAD65, glutamic acid decarboxylase 2; GAD67, glutamic acid decarboxylase 1; GAPDH, glyceraldehyde 3-phosphate dehydrogenase; NLGN, neuroligin; NR2A, N-methyl-D-aspartate receptor subunit 2A; NR2B, N-methyl-D-aspartate receptor subunit 2B; PSD95, postsynaptic density protein 95; PV, parvalbumin; VGAT, vesicular GABA transporter; VGULT, vesicular glutamate transporter.

### 2.4 Immunohistological analysis

Figures 1D,E show the timeline from MS or EE exposure to brain sampling for immunohistological analysis on PD 35. After measuring the body weight, each group was anesthetized with isoflurane between 13:00 and 16:00 h on PD 35 and intracardially fixed with 4% paraformaldehyde/phosphate-buffered saline (PBS) immediately after perfusion with medical-grade physiological saline to remove blood. The brains were removed from the skulls and post-fixed in 4% paraformaldehyde/PBS saline for 24 h at 4°C. Subsequently, after brain weight was measured, the fixed brains were soaked in 15% sucrose/phosphate buffer for 90 min at 4°C, followed by 30% sucrose/phosphate buffer for 18 h at 4°C for cryoprotection. The brains were then embedded in an optimal cutting temperature compound (Cat. No. 4583; Sakura Finetek, Tokyo, Japan) using dry ice and sectioned at 40 μm thickness using a cryostat (Leica, Wetzlar, Germany). The coronal slices in each brain area were selected using the rat brain atlas as a reference (Paxinos and Watson, 2007), and three slices from each brain region were used for analysis (SC: bregma −2.72, −2.88, and − 3.04 mm; mPFC: bregma 3.16, 3.32, and 3.48 mm). The sections were then immunostained via the free-floating method. Briefly, the sections were immersed in an antigen retrieval solution (HistoVT One: Cat. No. 06380–05; Nacalai Tesque, Kyoto, Japan) for 30 min at 70°C. They were then washed in PBS with 0.1% Tween 20 (PBST) three times for 5 min each at 22°C and then incubated in Blocking One Histo (Cat. No. 06349–64; Nacalai tesque) to block non-specific antibody reactions for 30 min at 22°C. After the sections were washed in PBST for 5 min at 22°C, they were incubated with primary antibodies and *Wisteria floribunda* agglutinin (WFA) diluted in PBS for 45 h at 4°C. Immunostaining was performed using a mouse monoclonal anti-PV antibody (1:2000; Cat. No. 195011, RRID:AB_2619882; Synaptic Systems, Göttingen, Germany), guinea pig polyclonal anti-neuronal nuclei (NeuN) antibody (1:3000; Cat. No. ABN90P, RRID:AB_2341095; Millipore, Massachusetts, United States), and biotinylated WFA (Final concentration: 2 μg/mL; Cat. No. BA-3101-2, EY laboratories, California, United States). Subsequently, the sections were washed in PBST three times for 5 min at 22°C and incubated with Alexa Fluor 488-conjugated goat anti-mouse IgG (1:1000; Cat. No. A-11029, RRID:AB_2534088; Thermo Fisher Scientific, Massachusetts, United States), CF633-conjugated goat anti-guinea pig IgG (1:3000; Cat. No. 20129, RRID:AB_10557034; Biotium, California, United States), and Texas Red streptavidin (1,2000; Cat. No. SA-5006-1, Vector laboratories, California, United States) diluted in PBS for 1.5 h at 22°C. The sections were then washed in PBST three times and in phosphate buffer twice for 5 min at 22°C and then mounted on low fluorescent glass slides (Cat. No. S0317; Matsunami Glass, Osaka, Japan). The sections were enclosed in cover glass (Cat. No. C024241; Matsunami Glass, Osaka, Japan) and sealed in VECTASHIELD Mounting Medium (Cat. No. H-1000; Vector Laboratories, California, United States). Fluorescent images were acquired using a confocal quantitative image cytometer (CQ1; Yokogawa, Tokyo, Japan) with a 20× objective lens. Each image was acquired as a z-stack (20 μm optical section thickness at 4.0 μm intervals) using an autofocus system with identical settings for laser power and exposure time between groups. The acquired images were processed for each section with maximum intensity projection and tiling using CellPathfinder (Yokogawa). The region of interest (ROI) for the dorsomedial prefrontal cortex (dmPFC) and ventromedial prefrontal cortex (vmPFC) were manually determined using the forceps minor of the corpus callosum as a guide by referencing the fluorescent staining image of NeuN with the Rat Brain Atlas (Paxinos and Watson, 2007). The dmPFC mainly includes the prelimbic cortex (PrL), and the vmPFC includes the infralimbic cortex (IL). Similarly, the ROI of the SC was manually determined using the hippocampus as a guide. The threshold was manually determined for each wavelength, and the number of positive cells was counted using CellPathfinder with identical settings. The detailed detection method for PV^+^ WFA^+^ cells is described in Supplementary Data 1. For the analysis that included the SC (Figure 1D, Experiment 2), the offset value was determined based on the intensity of PV expression in the SC (offset value = 600) because the PV expression in the SC was stronger than that in the dmPFC and vmPFC, and the low offset value determined based on the mPFC was too low for the SC, capturing extra synaptic puncta other than the cell body. Therefore, in this experiment, the number of PV^+^ cells in the dmPFC and vmPFC was undervalued but was evaluated in comparison with that of the SC. For analysis in the EE experiment (Figure 1E, Experiment 3), the offset value was determined based on the intensity of PV expression in the dmPFC to evaluate all PV^+^ interneurons as much as possible (offset value = 200).

### 2.5 Statistical analysis

All statistical analyses were performed using two-tailed tests in IBM SPSS Statistics for Windows, version 28.0 (IBM Corp., Armonk, New York, United States). Statistical significance was set at *value of p* <0.05. First, the Shapiro–Wilk test was performed to confirm the normality of all data, considering the sample size. The data between the two groups were analyzed via the Student’s *t*-test after confirming that the variances were equal via the Levene’s test. When variances were unequal, Welch’s *t*-test was performed. The Mann–Whitney *U*-test was performed to analyze non-normal distribution. If a significant difference in gene expression was observed between two groups, *q*-values were calculated using the Benjamini–Hochberg procedure to estimate the false discovery rate (FDR) among the results from PD 7 to PD 35. Significant difference was set at *value of p* <0.05 and 5% FDR (*q*-value <0.05). One-way analysis of variance (ANOVA) was conducted after confirming equal variances using the Levene’s test for the four groups in the EE experiment. Welch’s ANOVA was used when equal variance was not confirmed via the Levene’s test. The Kruskal–Wallis test was used for non-normally distributed data (the number of NeuN^+^ cells in the dmPFC). Subsequently, the Tukey–Kramer test was performed as a *post-hoc test* when significant differences were observed via one-way ANOVA. Supplementary Data 2 provides all the detailed statistical results.

## 3 Results

### 3.1 An enriched environment does not affect the MS-induced reduction of body and brain weights

The body weights in the MS group were lower than that in the MRC group (Table 2, PD7: *t*_(14)_ = 4.157, *p* < 0.001, PD14: *t*_(14)_ = 6.250, *p* < 0.001) and remained so after MS (Table 2, PD21: *t*_(14)_ = 10.268, *p* < 0.001, PD28: *z* = 3.361, *p* < 0.001, PD35: *t*_(14)_ = 6.610, *p* < 0.001). Likewise, the brain weights in the MS group were lower from PD 7 to PD 35 (Table 2, PD7: *t*_(14)_ = 4.269, *p* < 0.001, PD14: *t*_(14)_ = 5.678, *p* < 0.001, PD21: *t*_(14)_ = 7.460, *p* < 0.001, PD28: *z* = 2.731, *p* = 0.005, PD35: *z* = 3.153, *p* = 0.001). The EE showed no impact on the reduction in body and brain weight in the MS group on PD 35 (body weight: one-way ANOVA, *F*_(3. 22)_ = 37.380, *p* < 0.001; Tukey–Kramer *post-hoc* test, MRC vs. MS: *p* < 0.001, MRC vs. MS/EE: *p* < 0.001, MRC/EE vs. MS: *p* < 0.001, MRC/EE vs. MS/EE: *p* < 0.001, MS vs. MS/EE: *p* = 0.998) (brain weight: Welch’s ANOVA, *F*_(3. 11.238)_ = 21.682, *p* < 0.001; Games–Howell *post-hoc* test, MRC vs. MS: *p* = 0.001, MRC vs. MS/EE: *p* = 0.011, MRC/EE vs. MS: *p* < 0.001, MRC/EE vs. MS/EE: *p* = 0.009, MS vs. MS/EE: *p* = 1.000) (Table 3).

**Table 2:** Body and brain weights in the MRC and MS groups from PD 7 to PD 35.

	PD 7	PD 14	PD 21	PD 28	PD 35
Body weight (g)					
MRC	17.27 ± 0.63	33.62 ± 1.34	50.06 ± 1.80	87.31 ± 1.81	139.84 ± 4.01
MS	13.82 ± 0.54***	23.53 ± 0.91***	28.17 ± 1.14***	57.46 ± 4.46***	104.97 ± 3.42***
Brain weight (g)					
MRC	0.730 ± 0.014	1.280 ± 0.016	1.541 ± 0.021	1.648 ± 0.030	1.814 ± 0.011
MS	0.659 ± 0.009***	1.124 ± 0.022***	1.335 ± 0.018***	1.469 ± 0.029***	1.651 ± 0.021***

Data are expressed as mean ± SE. Brain weights were measured immediately after perfusion with saline for gene expression analysis. Both body and brain weights were analyzed using Student’s *t*-test, ***p < 0.001. PD, postnatal day; MRC, mother-reared control; MS, maternal separation.

**Table 3:** Body and brain weights in each group with or without enriched environment exposure on PD 35.

	MRC	MS	MRC/EE	MS/EE
Body weight (g)	161.46 ± 3.53	120.20 ± 3.57*^, #^	147.93 ± 2.02	119.37 ± 4.22*^, #^
Brain weight (g)	1.654 ± 0.020	1.510 ± 0.020*^, #^	1.660 ± 0.019	1.507 ± 0.029*^, #^

Data are expressed as mean ± SE. Brain weight was measured after perfusion and post-fixation with 4% paraformaldehyde/phosphate buffer saline for immunohistological analysis, unlike the data in Table 2. Body weight was analyzed using a post-hoc test (Tukey–Kramer test) after confirming significant differences by one-way analysis of variance (ANOVA). Brain weights were analyzed using a post-hoc test (Games–Howell test) after confirming significant differences by Welch’s ANOVA because equal variances could not be confirmed. *vs. MRC, *p* < 0.05; ^#^vs. MRC/EE, *p* < 0.05. EE, enriched environment; PD, postnatal day; MRC, mother-reared control; MS, maternal separation.

### 3.2 MS disrupts the gene expression of excitatory and inhibitory factors during the developmental period

**Figure 2:**
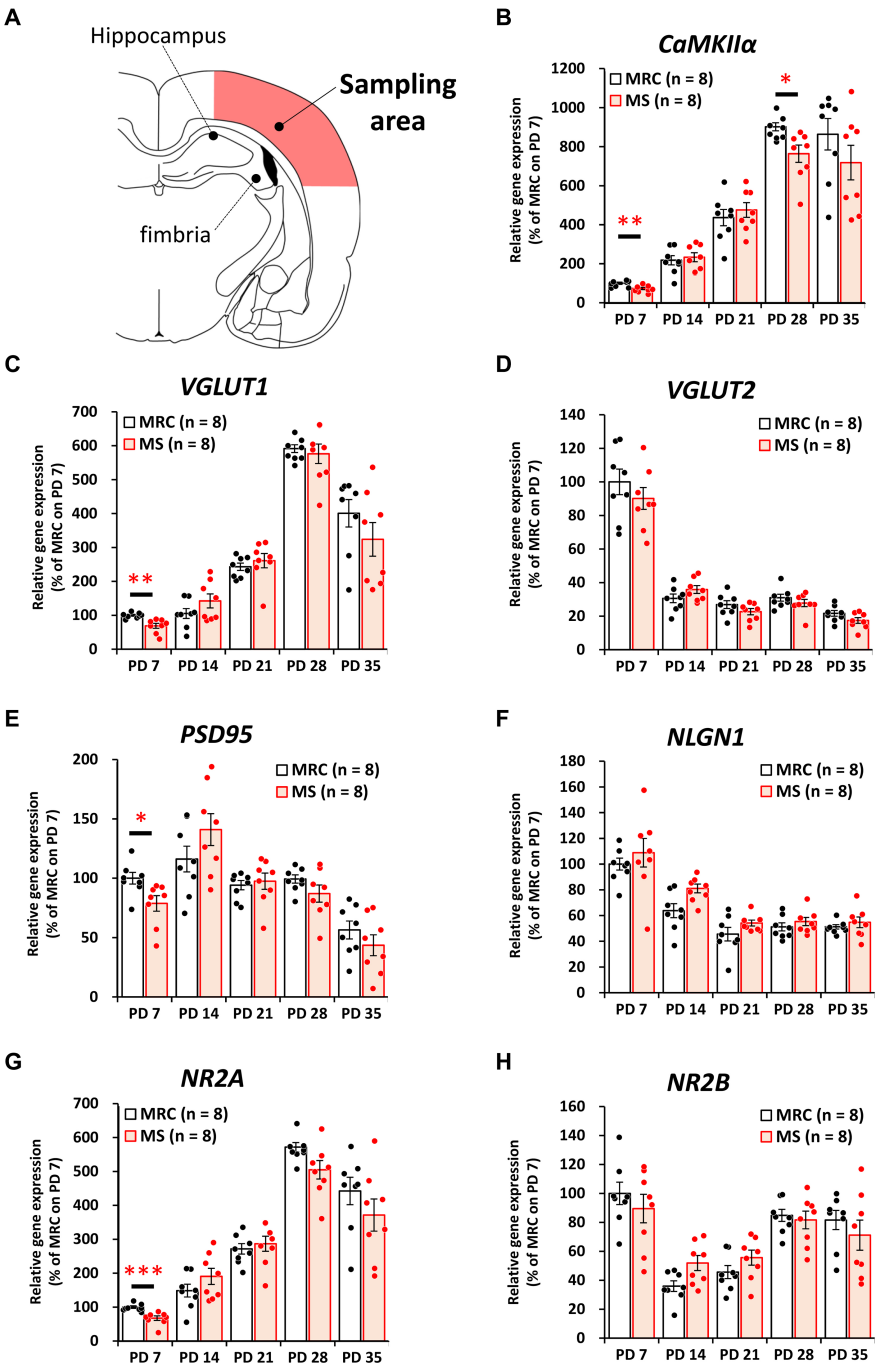
Influences of maternal separation (MS) on mRNA levels of various factors related to excitatory neurons in the sensory cortex. **(A)** A unilateral illustration of the analyzed brain area (red) and the indicator for sampling. **(B)** *CaMKIIα*: calcium/calmodulin-dependent protein kinase type II subunit alpha. **(C)** *VGLUT1*: vesicular glutamate transporter 1. **(D)** *VGLUT2*: vesicular glutamate transporter 2. **(E)** *PSD95*: postsynaptic density protein 95. **(F)** *NLGN1*: neuroligin 1. **(G)** *NR2A*: N-methyl-D-aspartate receptor (NMDAR) 2A subunits. **(H)** *NR2B*: NMDAR 2B subunits. The data were obtained from eight animals per group and are expressed as mean ± SE. Student’s *t*-test or Mann–Whitney *U*-test were used to determine statistically significant differences between the mother-reared control (MRC) and MS groups. When a significant difference was observed in gene expression between the two groups during PD 7–35, the *q*-value was evaluated among the results between PD 7–35 using 5% FDR (*q*-value <0.05) as the threshold. *p < 0.05, **p < 0.01, ***p < 0.001.

**Figure 3:**
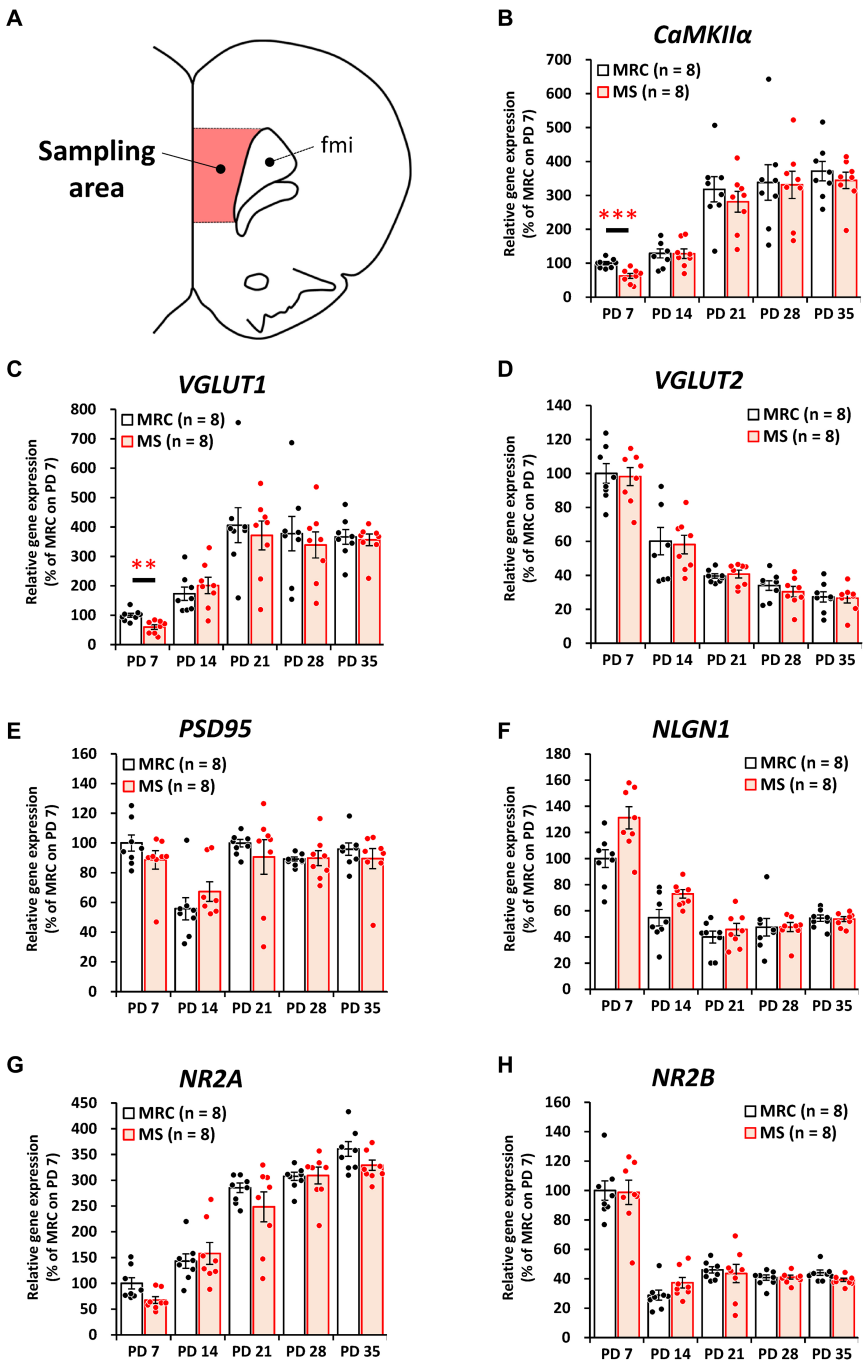
Influences of maternal separation (MS) on various factors related to excitatory neurons in the medial prefrontal cortex. **(A)** A unilateral illustration of the analyzed brain area (red) and an indicator for sampling. Fmi: forceps minor of the corpus callosum. **(B)** *CaMKIIα*: calcium/calmodulin-dependent protein kinase type II subunit alpha. **(C)** *VGLUT1*: vesicular glutamate transporter 1. **(D)** *VGLUT2*: vesicular glutamate transporter 2. **(E)** *PSD95*: postsynaptic density protein 95. **(F)** *NLGN1*: neuroligin 1. **(G)** *NR2A*: N-methyl-D-aspartate receptor (NMDAR) 2A subunits. **(H)** *NR2B*: NMDAR 2B subunits. The data were obtained from eight animals per group and are expressed as mean ± SE. Student’s *t*-test or Mann–Whitney *U*-test were used to determine statistically significant differences between the mother-reared control (MRC) and MS groups. When a significant difference was observed in gene expression between the two groups during PD 7–35, the *q*-value was evaluated among the results between PD 7–35 using 5% FDR (*q*-value <0.05) as the threshold. **p < 0.01, ***p < 0.001.

**Figure 4:**
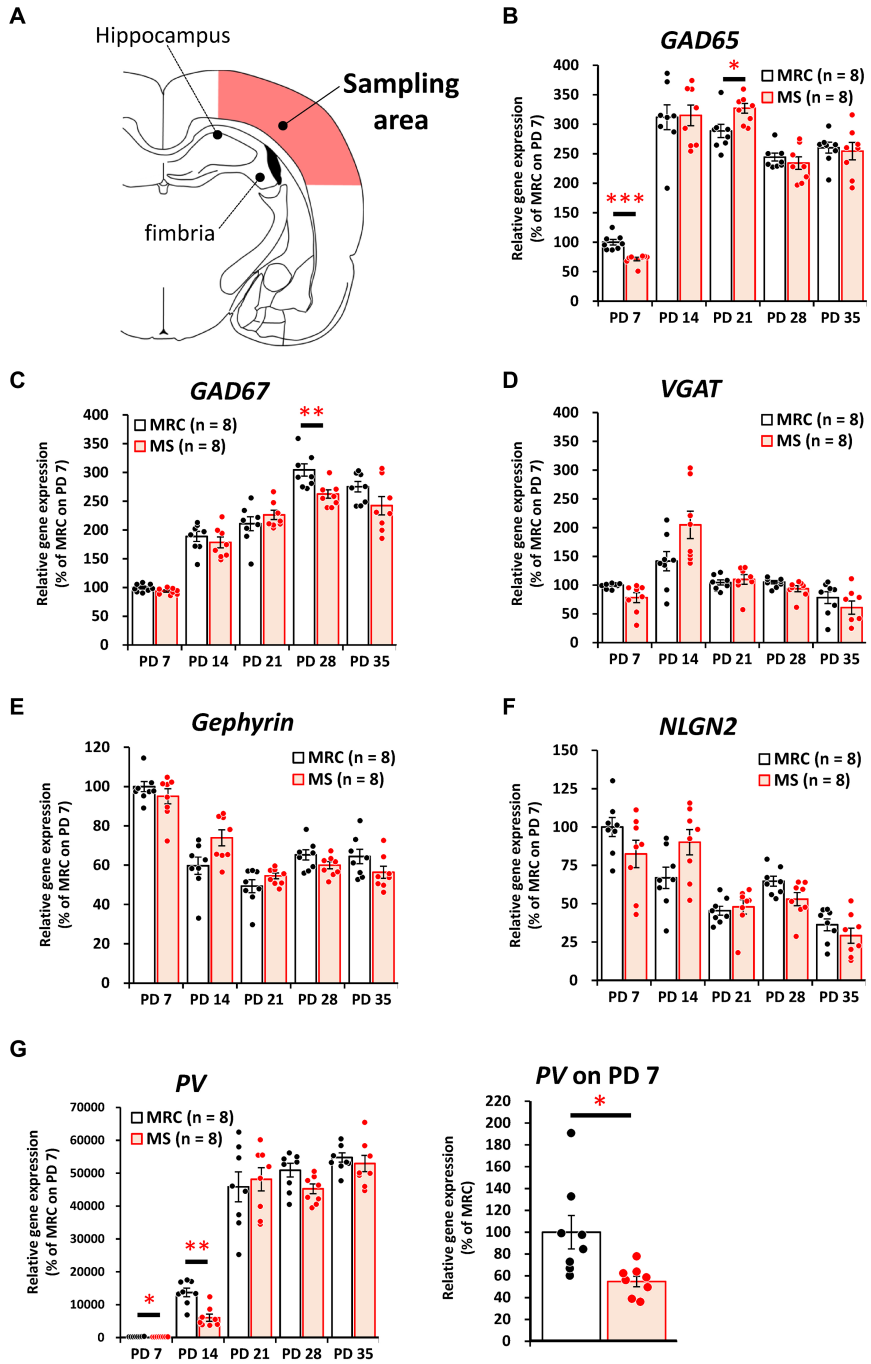
The influences of maternal separation (MS) on mRNA levels of various factors related to inhibitory neurons in the sensory cortex. **(A)** A unilateral illustration of the analyzed brain area (red) and an indicator for sampling (same area in Figure 2A). **(B)** *GAD65*: glutamic acid decarboxylase 2. **(C)** *GAD67*: glutamic acid decarboxylase 1. **(D)** *VGAT*: vesicular GABA transporter. **(E)** Gephyrin. **(F)** *NLGN2*: neuroligin 2. **(G)** *PV*: parvalbumin. Left, gene expression from PD 7 to PD 35; Right, enlarged graph on PD 7. The data were obtained from eight animals per group and are expressed as mean ± SE. Student’s *t*-test, Welch’s *t*-test, or Mann–Whitney *U*-test were used to determine statistically significant differences between the mother-reared control (MRC) and MS groups. When a significant difference was observed in gene expression between the two groups during PD 7–35, the *q*-value was evaluated among the results between PD 7–35 using 5% FDR (*q*-value <0.05) as the threshold. *p < 0.05, **p < 0.01, ***p < 0.001.

**Figure 5:**
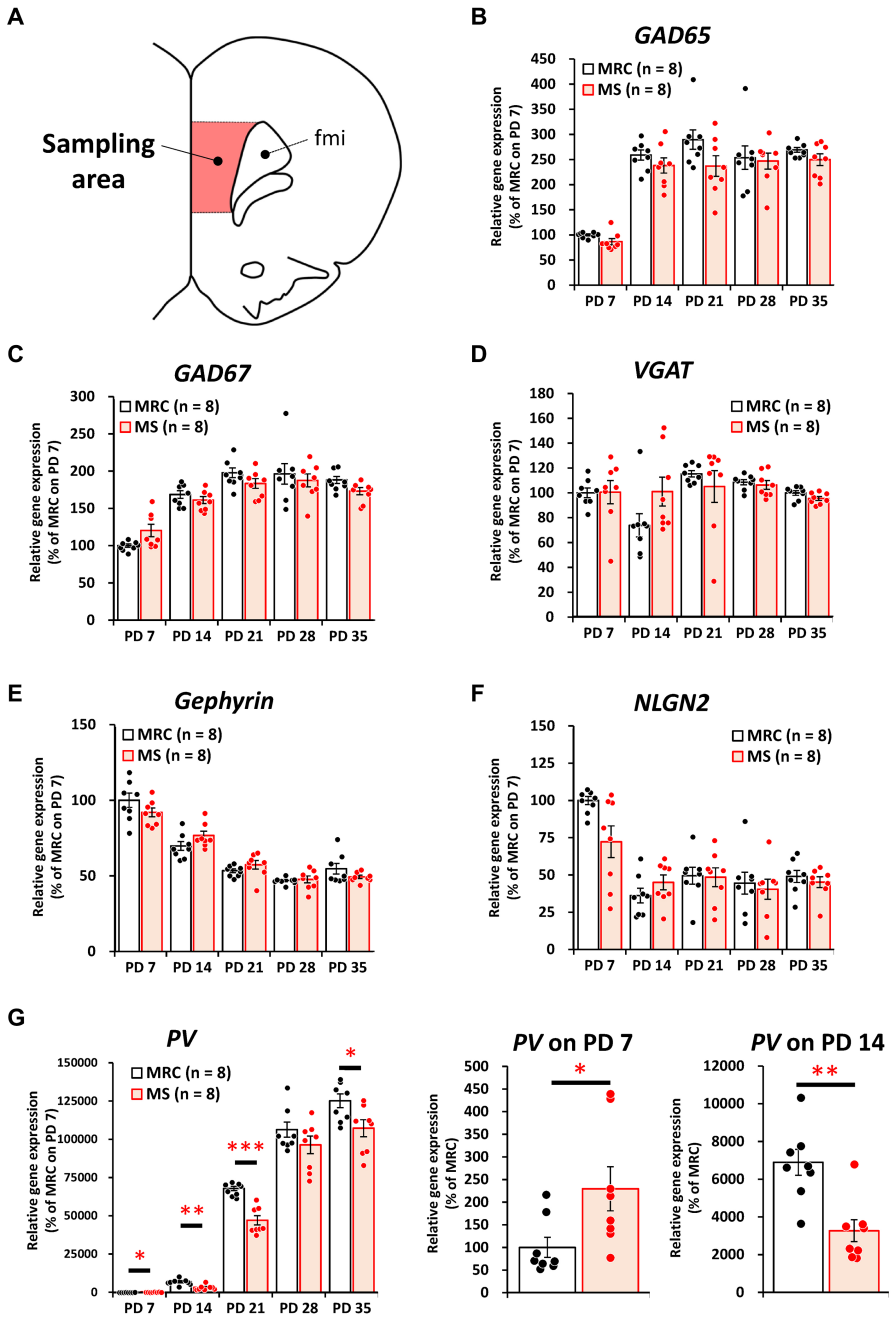
The influences of maternal separation (MS) on mRNA level of various factors related to inhibitory neurons in the medial prefrontal cortex. **(A)** A unilateral illustration of the analyzed brain area (red) and an indicator for sampling (Same area in Figure 3A). fmi: forceps minor of the corpus callosum. **(B)** *GAD65*: glutamic acid decarboxylase 2. **(C)** *GAD67*: glutamic acid decarboxylase 1. **(D)** *VGAT*: vesicular GABA transporter. **(E)** Gephyrin. **(F)** *NLGN2*: neuroligin 2. **(G)** *PV*: parvalbumin. Left, gene expression from PD 7 to PD 35; middle, enlarged graph on PD 7; right, enlarged graph on PD 7. The data were obtained from eight animals per group and are expressed as mean ± SE. Student’s *t*-test or Mann–Whitney *U*-test were used to determine statistically significant differences between the mother-reared control (MRC) and MS groups. When a significant difference was observed in gene expression between the two groups during PD 7–35, the *q*-value was evaluated among the results between PD 7–35 using 5% FDR (*q*-value <0.05) as the threshold. *p < 0.05, **p < 0.01, ***p < 0.001.

To evaluate how MS interferes with brain development, we examined the influences on the mRNA levels of excitatory and inhibitory factors in the SC and mPFC (Figures 2–5). Both regions showed significantly altered mRNA levels during the MS period, especially on PD 7. Specifically, among excitatory factors in the SC, MS significantly decreased the gene expression levels of *CaMKIIα* (Figure 2B, *t*_(14)_ = 3.152, *p* = 0.007, *q* = 0.035), vesicular glutamate transporter (*VGLUT*) 1 (Figure 2C, *t*_(14)_ = 3.962, *p* = 0.001, *q* = 0.005), postsynaptic density protein 95 (*PSD95*) (Figure 2E, *z* = 2.626, *p* = 0.007, *q* = 0.035), and N-methyl-D-aspartate receptor 2A subunit (*NR2A*) (Figure 2G, *t*_(14)_ = 4.248, *p* < 0.001, *q* = 0.004) on PD 7. In addition, only the gene expression level of *CaMKIIα* was significantly decreased in the MS group on PD 28 (Figure 2B: *t*_(14)_ = 2.818, *p* = 0.014, *q* = 0.035). In the mPFC, a similar tendency was observed, and MS significantly reduced the gene expression levels of *CaMKIIα* (Figure 3B, *t*_(14)_ = 4.204, *p* < 0.001, *q* = 0.004) and *VGLUT1* (Figure 3C, *t*_(14)_ = 3.869, *p* = 0.002, *q* = 0.010) on PD 7. In contrast, although the *q*-value ranged from 0.05 to 0.10, the gene expression of neuroligin1 (*NLGN1*) tended to increase during the MS period in the SC (Figure 2F, PD 14: *t*_(14)_ = 2.691, *p* = 0.018, *q* = 0.090) and mPFC (Figure 3F, PD 7: *t*_(14)_ = 2.881, *p* = 0.012 *q* = 0.060; PD14: *t*_(14)_ = 2.566, *p* = 0.022, *q* = 0.055).

Among the inhibitory factors in the SC, MS significantly decreased the gene expression levels of glutamic acid decarboxylase 2 (*GAD65*) (Figure 4B: PD 7, *z* = 3.361, *p* < 0.001, *q* = 0.001) and *PV* (Figure 4G: PD 7, *t*_(14)_ = 2.821, *p* = 0.014, *q* = 0.035; PD14, *z* = 3.046, *p* = 0.001, *q* = 0.005). After the MS period, a significantly decreased gene expression of glutamic acid decarboxylase 1 (*GAD67*) was observed on PD 28 (Figure 4C, *t*_(14)_ = 3.252, *p* = 0.006, *q* = 0.030). In contrast, increased gene expression of *GAD65* was observed on PD 21 (Figure 4B, *t*_(14)_ = 2.749, *p* = 0.016, *q* = 0.040). In the mPFC, *PV* gene expression in the MS group was significantly increased on PD 7 (Figure 5G, *z* = 2.310, *p* = 0.021, *q* = 0.035) but was decreased on PD 14 (Figure 5G, *z* = 2.836, *p* = 0.003, *q* = 0.008). In addition, the reduction in *PV* expression in the MS group was sustained until PD 21 (Figure 5G, *t*_(10.003)_ = 6.125, *p* < 0.001, *q* < 0.001) and transiently increased to a level equal to that in the MRC group on PD 28 (Figure 5G, *z* = 0.630, *p* = 0.574). However, it decreased again on PD 35 (Figure 5G, *t*_(14)_ = 2.526, *p* = 0.024, *q* = 0.030).

These results suggest that MS disrupts both excitatory and inhibitory factors. Moreover, some of these influences persisted even after the MS period, and especially the effects of MS are likely to persistently affect PV^+^ interneurons in the mPFC.

### 3.3 MS decreases the number of PV^+^ interneurons in the vmPFC on PD 35

**Figure 6:**
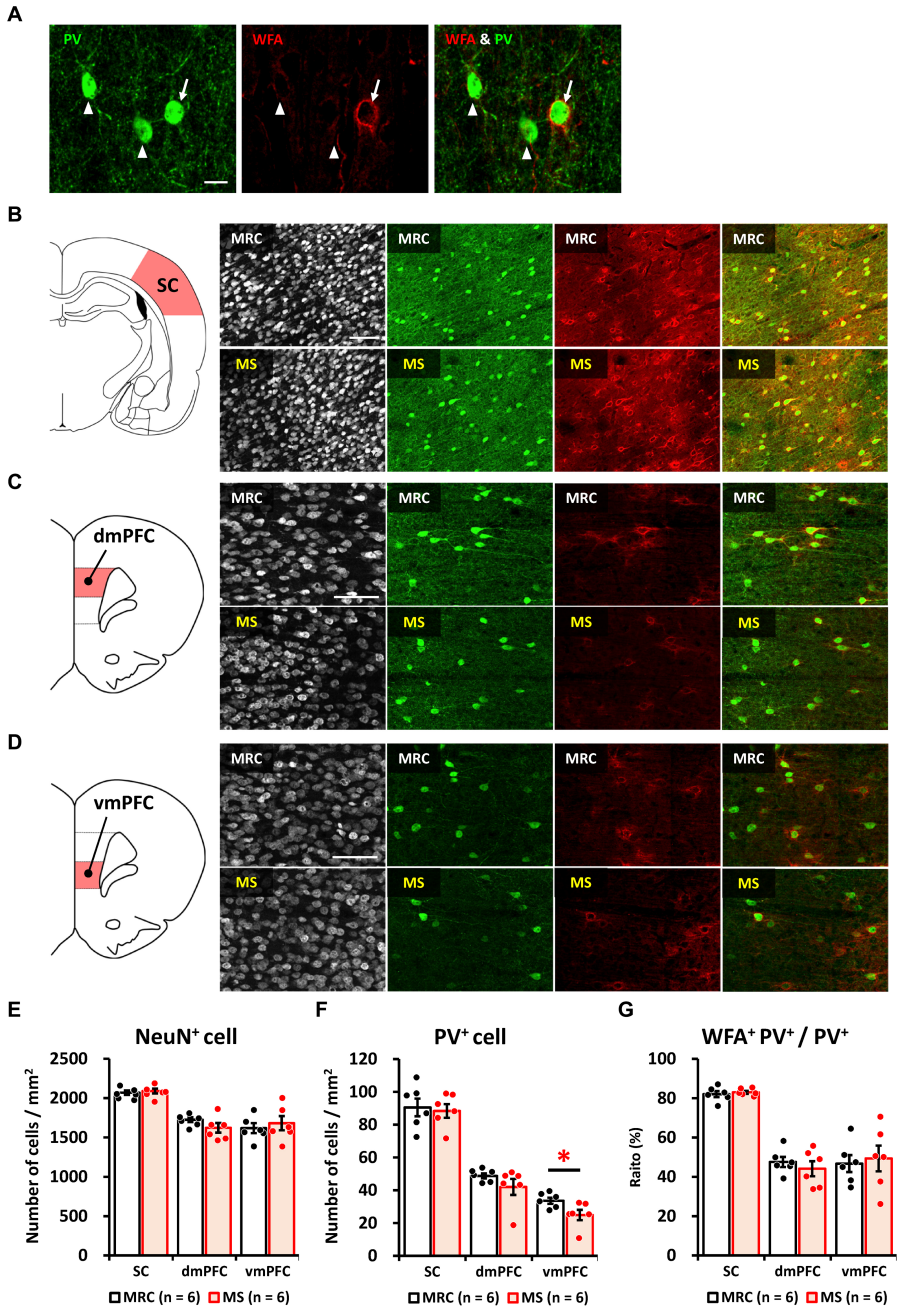
Maternal separation (MS) decreased the number of parvalbumin (PV)^+^ interneurons in the ventromedial prefrontal cortex (vmPFC) on PD 35. **(A)** Immunostaining image of parvalbumin (*PV*)^+^, *Wisteria floribunda* agglutinin (WFA)^+^, and WFA^+^ PV^+^ interneurons. Arrow: WFA^+^ PV^+^ interneuron. Arrowhead: PV^+^ interneuron without WFA. Scale bar = 20 μm. **(B)** From left to right, an illustration of the region of interest (ROI) for the sensory cortex (SC) and immunostaining images of NeuN, PV, and WFA, and overlapped images (PV & WFA) in both groups. Scale bar = 100 μm. **(C)** From left to right, an illustration of the region of interest (ROI) for the dorsomedial prefrontal cortex (dmPFC), and immunostaining images of neuronal nuclear protein (NeuN), PV, and WFA, and overlapped images (PV & WFA) in both groups. Scale bar = 100 μm. **(D)** From left to right, an illustration of the ROI for the ventromedial prefrontal cortex (vmPFC); immunostaining images of NeuN, PV, and WFA, and overlapped images (PV and WFA) in both groups. Scale bar = 100 μm. **(E)** Numbers of NeuN^+^ cells in each brain region in the MRC and MS groups. **(F)** Number of PV^+^ cells in each brain region of both groups. **(G)** The ratio of WFA^+^ PV^+^ interneurons to PV^+^ interneurons in each brain region of both groups. The data were obtained from six animals per group and are expressed as mean ± SE. Student’s *t*-test or Mann–Whitney *U*-test were used to determine statistically significant differences between the mother-reared control (MRC) and MS groups (*p < 0.05). Contrast adjustment of images **(B,C,D)** was performed using identical settings based on the SC. Please see Supplementary Data 3 for the overall image at low magnification.

Based on the results of gene expression analysis, we focused on how MS affects the development of PV^+^ inhibitory neurons and evaluated the number of PV^+^ cells, including PNN formation surrounding PV^+^ cells (Figure 6A), on PD 35. There were no significant changes in the numbers of NeuN^+^ cells in the SC (Figures 6B,E), dmPFC (Figures 6C,E), or vmPFC (Figures 6D,E). The number of PV^+^ cells was also unchanged in the SC (Figures 6B,F) and dmPFC (Figures 6C,F) between the two groups, whereas that in the vmPFC of the MS group was significantly lower than that of the MRC group (Figures 6D,F, *t*_(10)_ = 2.375, *p* = 0.039). The ratios of WFA^+^ PV^+^ to PV^+^ interneurons did not change in the SC (Figures 6B,G), dmPFC (Figures 6C,G), or vmPFC (Figures 6D,G).

These results indicate that the impact of MS on PV^+^ inhibitory neurons persists in the vmPFC but not in the SC. In addition, the results are similar to those after maturation in our previous study (Ohta et al., 2020), indicating that the number of PV^+^ interneurons in the MS group was already reduced on PD 35 and that the reduction is long-lasting. Moreover, the results of the WFA suggest that the reduction in PV^+^ interneuron number in the MS group is not attributable to changes in the critical period due to early or delayed development.

### 3.4 EE after weaning rescues the MS-induced reduction of PV^+^ interneuron numbers in the vmPFC before weaning

**Figure 7:**
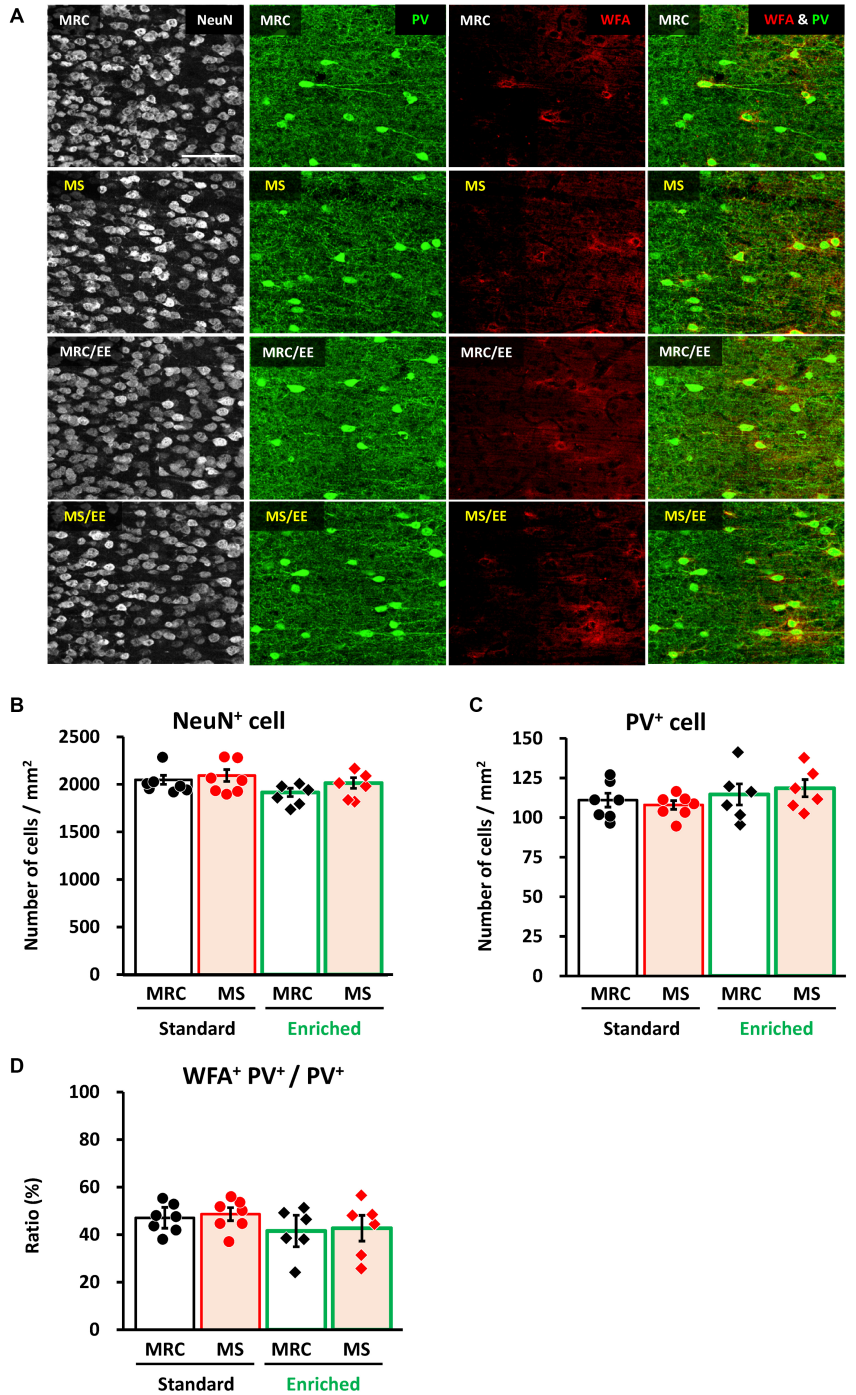
An enriched environment had little impact on the number of parvalbumin (PV)^+^ interneurons in the dorsomedial prefrontal cortex (dmPFC) on PD 35. **(A)** From left to right: immunostaining images of neuronal nuclear protein (NeuN), parvalbumin (PV), and *Wisteria floribunda* agglutinin (WFA), and overlapped images (PV & WFA) in each group. **(B)** Number of NeuN^+^ cells in each group. **(C)** Number of PV^+^ interneurons in each group. **(D)** Ratio of WFA^+^ PV^+^ to PV^+^ interneurons in each group. The data were obtained from seven animals per group in a standard environment and six animals per group in an enriched environment. Data are expressed as mean ± SE. After one-way or Welch’s analysis of variance (ANOVA), *post-hoc* tests (Tukey–Kramer or Games–Howell tests) were used to determine statistically significant differences between each group. MRC: mother-reared control, MS: maternal separation. Contrast adjustment of images **(A)** was performed using identical settings based on the dmPFC, unlike in Figure 6. Please see Supplementary Data 4 for the overall image at low magnification.

**Figure 8:**
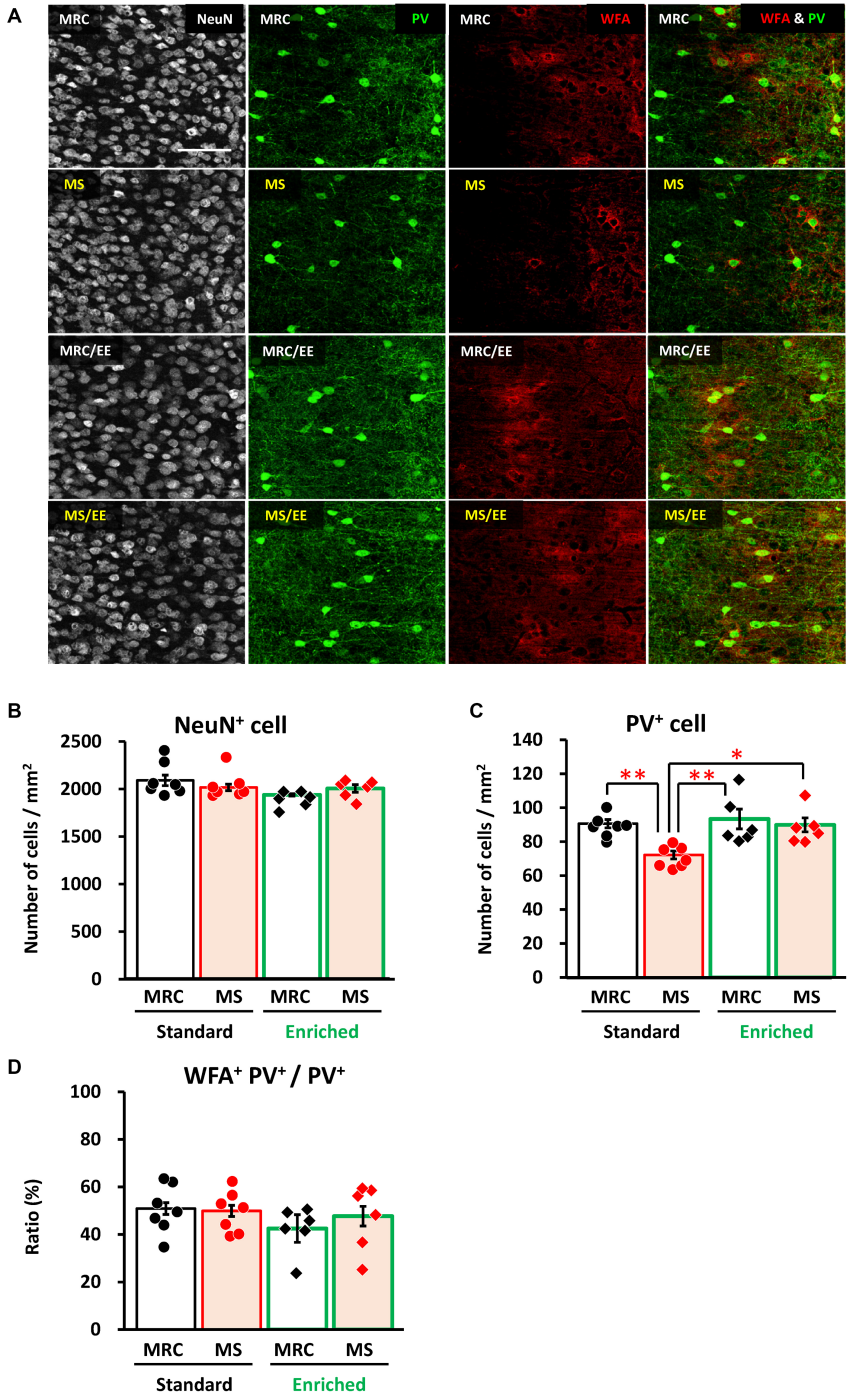
An enriched environment restored the maternal separation (MS)-induced reduction of parvalbumin (PV)^+^ interneurons in the ventromedial prefrontal cortex on PD 35. **(A)** From left to right: immunostaining images of neuronal nuclear protein (NeuN), parvalbumin (PV), and *Wisteria floribunda* agglutinin (WFA), and overlapped images (PV & WFA) in each group. **(B)** Number of NeuN^+^ cells in each group. **(C)** Number of PV^+^ interneurons in each group. **(D)** Ratio of WFA^+^ PV^+^ to PV^+^ interneurons in each group. The data were obtained from seven animals per group in a standard environment and six animals per group in an enriched environment. Data are expressed as mean ± SE. After one-way or Welch’s analysis of variance (ANOVA), *post-hoc* tests (Tukey–Kramer or Games–Howell tests) were used to determine statistically significant differences between each group. *p < 0.05, **p < 0.01. MRC: mother-reared control. Contrast adjustment of images **(A)** was performed using identical settings based on the dorsomedial prefrontal cortex (dmPFC), unlike in Figure 6. Supplementary Data 4 shows the overall image at low magnification.

Finally, we investigated whether an EE after the MS period improved the effects of MS on the mPFC. In the dmPFC, the enrichment environment did not have a significant impact on the number of NeuN^+^ (Figures 7A,B), PV^+^ (Figures 7A,C), or the ratio of WFA^+^ PV^+^ cells (Figures 7A,D). Similarly, in the vmPFC, we observed no alterations in the number of NeuN^+^ (Figures 8A,B) or the ratio of WFA^+^ PV^+^ cells (Figures 8A,D). However, the EE restored the reduction in PV^+^ interneurons in the vmPFC of the MS group to the same level as that in the MRC group (Figures 8A,C, one-way ANOVA, *F*_(3. 22)_ = 6.927, *p* = 0.002; Tukey–Kramer *post-hoc* test, MRC vs. MS: *p* = 0.008, MRC/EE vs. MS: *p* = 0.003, MS/EE vs. MS: *p* = 0.015). These results indicate that the influence of MS on the mPFC during early brain development can be reversed depending on the subsequent environment, at least before the end of the critical period in the mPFC.

## 4 Discussion

In the present study, MS interrupted the gene expression of excitatory and inhibitory factors related to enzymes, presynapses, postsynapses, and synaptic cell adhesion in the SC and mPFC. After the MS period, some of these factors were only slightly altered, whereas the reduction in PV expression persisted in the mPFC. Moreover, the number of PV^+^ interneurons decreased in the mPFC but not in the SC. In contrast, the ratio of PNN formation surrounding PV^+^ interneurons was unchanged in both regions, which indicates that MS decreased the number of PV^+^ interneurons in the mPFC but did not affect their maturational state around critical period closure. Moreover, the EE after the MS period restored the PV^+^ interneuron number in the mPFC. This result suggests that nurturing environment and experience during not only the brain growth spurt but also the critical period is important for the development of PV^+^ interneurons in the mPFC and that the influences of early adverse experience on the mPFC can be reversed by the subsequent environment.

In this study, MS caused a decrease in body and brain weights on PD 7, which lasted until PD 35. Moreover, an EE after the MS period did not restore the body and brain weights, which are very robust influences. In a previous study, we confirmed using a dynamic nutritional index that the MS group is not undernourished (Ohta et al., 2014). In addition, breast milk was still present in the stomach and small intestine of the MS group during brain sampling. Therefore, the low body and brain weights in the MS group were unlikely to have been caused by starvation. Meanwhile, MS clearly deprived contact with the dam, including the opportunity of feeding, from the pups. Considering the serum corticosterone level in the MS group increases during MS despite the lack of stimulation (Ohta et al., 2014), such deprivation is likely to cause anxiety and emotional stress in the pups. In addition, the disruption of feeding patterns might upset the circadian rhythm during MS (Reite et al., 1982). We believe these impacts may cause body and brain weight losses; however, further studies are needed to elucidate the mechanism.

The gene expression level of most factors, except for *PV* in the mPFC, showed no long-lasting changes until PD 35 between the two groups. In both groups, excitatory (*CaMKIIα, VGLUT1, and NR2A*) and inhibitory (*GAD65* and *GAD67*) factors tended to increase at the same extent during PD 7–35. In particular, the results of *NR2A* and N-methyl-D-aspartate receptor 2B subunit *(NR2B)* show no changes in the switching of NMDA receptors (Liu et al., 2004), which has an important role in synaptic formation (Gambrill and Barria, 2011) and neural plasticity (Franchini et al., 2019) during the early developmental period. Since there are no changes in the long-lasting variation of gene expression related to excitatory and inhibitory neurons during brain development, MS might not cause a permanent delay in synaptic formation. However, MS transiently disturbed the gene expression of some excitatory and inhibitory neuronal factors, particularly on PD 7. The disrupted expression on PD 7 can be attributed to a reduction in brain-derived neurotrophic factor (*BDNF*) levels. In this study, we confirmed the decreased gene expression of *BDNF* on PD 7 in the SC and mPFC (Supplementary Data 5). In addition, we previously reported that MS attenuates both gene and protein levels of BDNF on PD 7 only in the mPFC (Tenkumo et al., 2020). Furthermore, the correlation between the expression of many factors associated with excitatory and inhibitory neurons and BDNF expression was higher in both regions on PD 7 than that on subsequent PDs. Reduced factors in both or either region of the MS group, such as *CaMKIIα*, *VGLUT1*, *PSD95*, *NR2A*, *GAD65*, and *PV,* strongly correlated with *BDNF* expression on PD 7 (Supplementary Data 6). In contrast, gene expression of *NLGN1* was not correlated with *BDNF* expression but tended to increase on PD 7. These results may indicate a mismatched developmental state of neural network formation, particularly primary synaptogenesis, via reduced BDNF signaling in the MS group on PD 7. BDNF plays a crucial role in synaptogenesis and dendritic arbor growth in both excitatory and inhibitory neurons (Cohen-Cory et al., 2010). The first and second postnatal weeks are a very important period for mPFC function after maturation. During this period, dendritic length/branching and spine density rapidly increase in the pyramidal neurons in layers 3 and 5 of the mPFC (Briner et al., 2011; Kroon et al., 2019). Transient excess activation of the mPFC during PD 7–10 causes excitatory and inhibitory imbalances, low working memory, and cognitive dysfunction observed around PD 40 (Bitzenhofer et al., 2021). In addition, 24 h of MS on PD 9 decreases inhibitory neurons in the mPFC (Poleksic et al., 2021) and impairs mPFC-related functions, such as cognition and flexibility, (Janetsian-Fritz et al., 2018; Poleksic et al., 2021) after maturation. Considering that PV^+^ interneurons in the vmPFC permanently decreased until PD 35 after MS ended, the first and second postnatal weeks in rodents are likely when the mPFC is vulnerable to adverse stimuli. Our results suggest that the reduction in BDNF expression in the mPFC by MS can disturb neural circuit formation from the first to the second weeks of age, leading to a persistent imbalance of excitatory and inhibitory neurons after maturation, as reported previously (Ohta et al., 2020; Chen et al., 2021). In contrast, in the SC, only a few factors related to excitatory and inhibitory neurons were reduced on PD28, with no long-lasting influences through PD 35, unlike in the mPFC, at least in this study. However, earlier studies indicated that MS causes abnormal sensitivity to somatosensory stimuli after maturation (Takatsuru et al., 2009; Prusator and Greenwood-Van Meerveld, 2016). Considering that the first and second postnatal weeks in rodents correspond to the critical period of the SC (Lendvai et al., 2000; Lo et al., 2017; Reh et al., 2020), the disruption of factors related to excitatory and inhibitory neurons in our results might subsequently cause excitatory and inhibitory imbalances later in life, leading to hyperesthesia. However, as a limitation of this study, factors related to excitatory and inhibitory neurons, except for PV, have been evaluated only in terms of gene expression. Further studies are needed to clarify the impact of MS on excitatory/inhibitory balance, including its relationship with BDNF signaling.

In the present study, the number of PV^+^ interneurons was reduced on PD 35 in the vmPFC but not in the dmPFC. Given the lower body and brain weights in the MS group, the decrease in PV^+^ cells might be attributed to developmental delay. However, the levels of factors that increase or decrease with development, such as *VGLUT1* and *VGLUT2*, transiently changed but were not consistently delayed throughout development until PD 35. Moreover, as PNN formation surrounding PV^+^ cells was unchanged, it appeared that the critical period of the mPFC was not delayed by MS, at least on PD35. In our previous study, this reduction in PV^+^ cells did not reach the level observed in the MRC group at 8 weeks of age (Ohta et al., 2020). Therefore, delayed brain development was not likely to have led to the reduction in PV^+^ interneurons in the vmPFC. The mechanism by which MS affects the development of PV^+^ interneurons in the vmPFC is unclear. However, the reduction of BDNF by MS may be among the causes. BDNF signaling increases PV expression (Patz et al., 2004) and accelerates the maturation of PV^+^ neurons (Huang et al., 1999). Du et al. reported that BDNF heterozygous mice exhibited decreased PV^+^ cell density in the IL, which is the ventral part of the mPFC, but not in the PrL and cingulate cortex, which are the dorsal part of the mPFC, at 6 and 12 weeks of age (Du et al., 2018). These results suggest that the vmPFC is most sensitive to BDNF in the mPFC. However, considering that BDNF signaling also accelerates interneuron migration from the medial ganglionic eminence during the embryonic day (Polleux et al., 2002), the reduction in PV^+^ cell density in the vmPFC may be due to delayed migration by attenuated BDNF signaling during the embryonic stage in the case of *BDNF* transgenic animals, unlike our results. Another reason is that the maturation of the vmPFC may be particularly slow compared to other brain regions. We confirmed that PV^+^ cell density was unchanged not only in the SC but also in the lateral/basolateral amygdala (BLA) and dorsal hippocampus (Supplementary Data 7), and the maturation of PV^+^ interneurons in these areas occurred earlier than that in the mPFC (Reh et al., 2020). Moreover, some studies have indicated that PV^+^ cell density and maturation in the vmPFC are delayed more than in the dmPFC (Ueno et al., 2017; Richardson et al., 2021). Therefore, the immature vmPFC may be more susceptible than the dmPFC to the attenuation of BDNF expression around PD 7, as observed in this and previous studies (Tenkumo et al., 2020). To clarify the difference in influence between the dorsal and ventral parts of the mPFC, further detailed analysis is required regarding the effect of time-specific BDNF on the mPFC.

The vmPFC is strongly associated with social recognition. In human studies, patients with vmPFC damage have shown deficits in emotional and cognitive empathy (Barrash et al., 2000; Shamay-Tsoory et al., 2009). Chemogenetic research in mice has revealed different functions between the dorsal and ventral parts of the mPFC for social behavior, and the projection from the IL to the BLA (IL-BLA) is activated more than that from the PrL to the BLA (PrL-BLA) during social behavior (Huang et al., 2020). In addition, Huang et al. reported that IL-BLA projections promote social contact in contrast to PrL-BLA projections. A human study on self-referential thought also indicated that the dmPFC is involved in mentalizing dissimilar others, whereas the vmPFC is involved in mentalizing similar others (Mitchell et al., 2006). The results of these studies suggest that the mutual relationship between the dmPFC and vmPFC is important for social recognition, such as the judgment of unfamiliar and familiar others. Moreover, projections from the IL to the nucleus accumbens shell play an important role in social recognition, and chemogenetic inhibition of this projection impairs social recognition without a deficit in social preference (Park et al., 2021). As PV^+^ interneurons in the mPFC play a crucial role in the formation of gamma oscillations related to social recognition and working memory (Cao et al., 2018; Bitzenhofer et al., 2021; Guan et al., 2022), a reduction in PV^+^ cells in the vmPFC is likely associated with the deficits observed in our previous studies (Ohta et al., 2020).

We previously reported that the reduction in PV^+^ interneurons in the vmPFC persisted after maturation (Ohta et al., 2020); however, an EE after MS restored this reduction to the level of that in the MRC group. Thus, this reduction is not irreversible, and there is room for recovery until the critical period of closure of the mPFC. We observed in the present study that *PV* gene expression increased rapidly in the mPFC from PD 14 to PD 21 but did not end on PD 21 as in the SC. It also increased rapidly between PD 21 and PD 35 after the MS period. Similarly, some studies have reported that the number of PV^+^ cells in the dmPFC and vmPFC rapidly increases from 3 to 5 weeks of age, unlike that in the BLA (Gildawie et al., 2020; Richardson et al., 2021). Considering that interneurons, which express PV with maturity as fast-spiking neurons, end their tangential and radial migration before PD 21 (Larsen et al., 2019; Warm et al., 2021), it is unlikely that EEs produce new PV^+^ interneurons to increase the absolute number. The dmPFC showed no increase in the number of PV^+^ interneurons beyond the level of the MRC group grown in the laboratory environment after exposure to an EE, and the reduction of PV^+^ cells by MS was restored to the level of the MRC group only in the vmPFC. Therefore, we believe that the EE caused experience-dependent enhancement of maturation in inhibitory neurons that are still immature and lack sufficient PV expression due to exposure to MS, leading to an increase in the number of PV^+^ interneurons. In addition, the impacts of MS are not limited to only the exposure period; rather, exposure subsequently disturbs the development of inhibitory neurons and neural circuit formation in the mPFC during the critical period. Our results showed that the *PV* gene expression in the MS group nearly reached the level of that in the MRC group once, but the gap widened again on PD 35. Likewise, *GAD67* gene expression did not change between PD 14 and PD 28 but was reduced on PD 35 despite MS ending on PD 21. These sustained effects of MS exposure are likely to lead to excitatory and inhibitory imbalances in the mPFC after maturation, as we reported previously (Ohta et al., 2020). An EE until the end of the critical period in the mPFC may have significance in preventing subsequent functional abnormalities by rescuing such persistent adverse effects in the early stage.

In the present study, we clarified that an EE recovered the MS-induced reduction of PV^+^ interneurons in the vmPFC; however, whether this recovery subsequently leads to the restoration of excitatory and inhibitory imbalance and abnormality of mPFC-related behaviors observed after maturation is unclear. In the mPFC, projections from or to other brain areas, such as the BLA and nucleus accumbens, after PD 35 gradually increase until adulthood (Cunningham et al., 2002; Brenhouse et al., 2008). An earlier study has reported that a social enriched environment, in which six animals were housed together after weaning to 9 weeks of age, enhanced social recognition (Gabriel et al., 2020). However, it is poorly understood whether an EE only during a critical period improves adverse influences by MS at the behavioral level. The results of the present study suggest that from a neuroscientific perspective, the influences of an adverse environment until the toddler stage of development are not necessarily irreversible and may be partially restored, at least in the mPFC, depending on the environment before preadolescence. Given that various functions related to the mPFC, especially empathy, are important for humans in society, our findings could lead to the development of care for children who have been exposed to abuse that impairs these functions.

## Data availability statement

The raw data supporting the conclusions of this article will be made available by the authors, without undue reservation.

## Ethics statement

The animal study was approved by the Animal Care and Use Committee for Kagawa University. The study was conducted in accordance with the local legislation and institutional requirements.

## Author contributions

KI: Conceptualization, Formal analysis, Investigation, Visualization, Writing – original draft, Validation. KO: Conceptualization, Formal analysis, Funding acquisition, Investigation, Writing – review & editing, Writing – original draft. HU: Investigation, Writing – review & editing. CA: Investigation, Writing – review & editing. KH: Investigation, Writing – review & editing. SS: Conceptualization, Formal analysis, Writing – review & editing. KW: Conceptualization, Formal analysis, Investigation, Writing – review & editing. HO: Formal analysis, Investigation, Writing – review & editing. HK: Investigation, Writing – review & editing. SN: Writing – review & editing, Conceptualization, Investigation. KK: Writing – review & editing, Conceptualization, Investigation. TM: Funding acquisition, Project administration, Supervision, Writing – review & editing. TK: Funding acquisition, Project administration, Supervision, Writing – review & editing.
