# Supplementary material for: An enriched environment ameliorates the reduction of parvalbumin-positive interneurons in the medial prefrontal cortex caused by maternal separation early in life
Source: Front Neurosci. 2024 Jan 16;17:1308368. doi: 10.3389/fnins.2023.1308368 (PMC10825025; doi:10.3389/fnins.2023.1308368)
Supplement: Supplementary file 1 [file Data_Sheet_1.docx]

# *Supplementary Material*

# Supplemental Data 1: PV^+^ WFA^+^ Interneuron Analysis

Parvalbumin (PV)- or *Wisteria floribunda* agglutinin (WFA)-immunostained images were processed and counted using CellPathfinder (Yokogawa, Tokyo, Japan). PV^+^ regions (Fig. S1A) were binarized using the same settings as for the analysis of PV^+^ cell number (Fig. S1B). After removing small puncta by size filter, images of PV^+^ interneurons were dilated using a 5 μm diameter setting (Fig. S1C). WFA^+^ regions (Fig. S1D) were also binarized (Fig. S1E), and detected (Fig. S1F). The positive regions (Fig. S1C and S1F) were merged, and overlapping areas exceeding a certain range were counted as PV^+^ WFA^+^ interneurons (Fig. S1G).

**
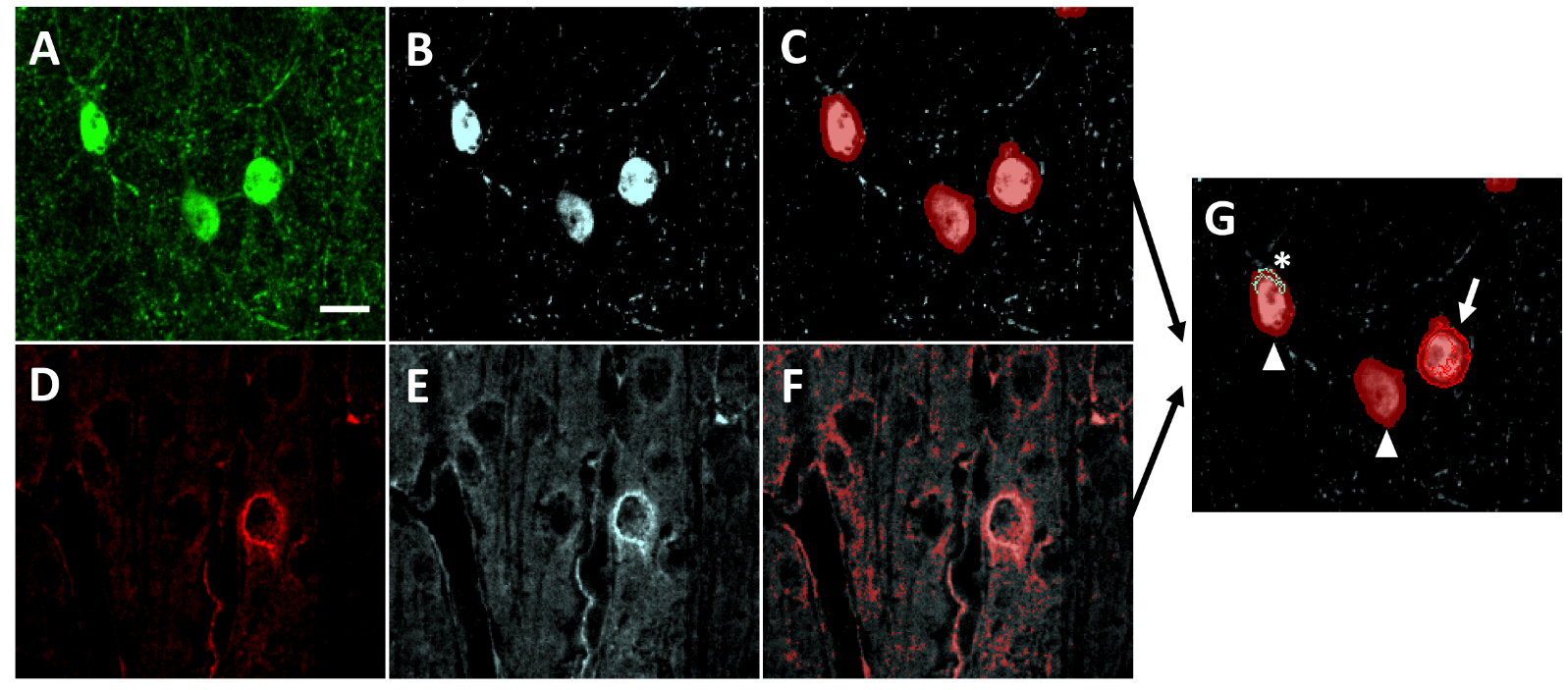
**

**Figure S1.** Images of PV^+^ WFA^+^ interneuron analysis. (A) Immunostaining image of a PV^+^ region. Scale bar = 20 μm. (B) Binarized image of a PV^+^ region. (C) Dilated image of PV^+^ interneurons after removing puncta. (D) Immunostaining image of a WFA^+^ region. (E) Binarized image of a WFA^+^ region. (C) Image of a detected WFA^+^ area. (G) Colored line: overlapping area of (C) and (F). Asterisk: uncounted area in PV^+^ WFA^+^ owing to an area less than the set criteria (>50 µm^2^), arrow: PV^+^ WFA^+^ interneuron, arrowhead: PV^+^ WFA^-^ interneuron.

**Supplemental Data 2: Statistical Analysis Results**

**Table S1.** Results of statistical analysis of data in Table 2.

**Table S2.** Results of statistical analysis of data in Table 3.

**Table S3.** Results of statistical analysis of data in Figure 2.

**Table S4.** Results of statistical analysis of data in Figure 3.

**Table S5.** Results of statistical analysis of data in Figure 4.

**Table S6.** Results of statistical analysis of data in Figure 5.

**Table S7.** Results of statistical analysis of data in Figure 6.

**Table S8.** Results of statistical analysis of data in Figure 7.

**Table S9.** Results of statistical analysis of data in Figure 8.

# Supplemental Data 3: Low-magnification Immunostaining Images from Figure 6.

**
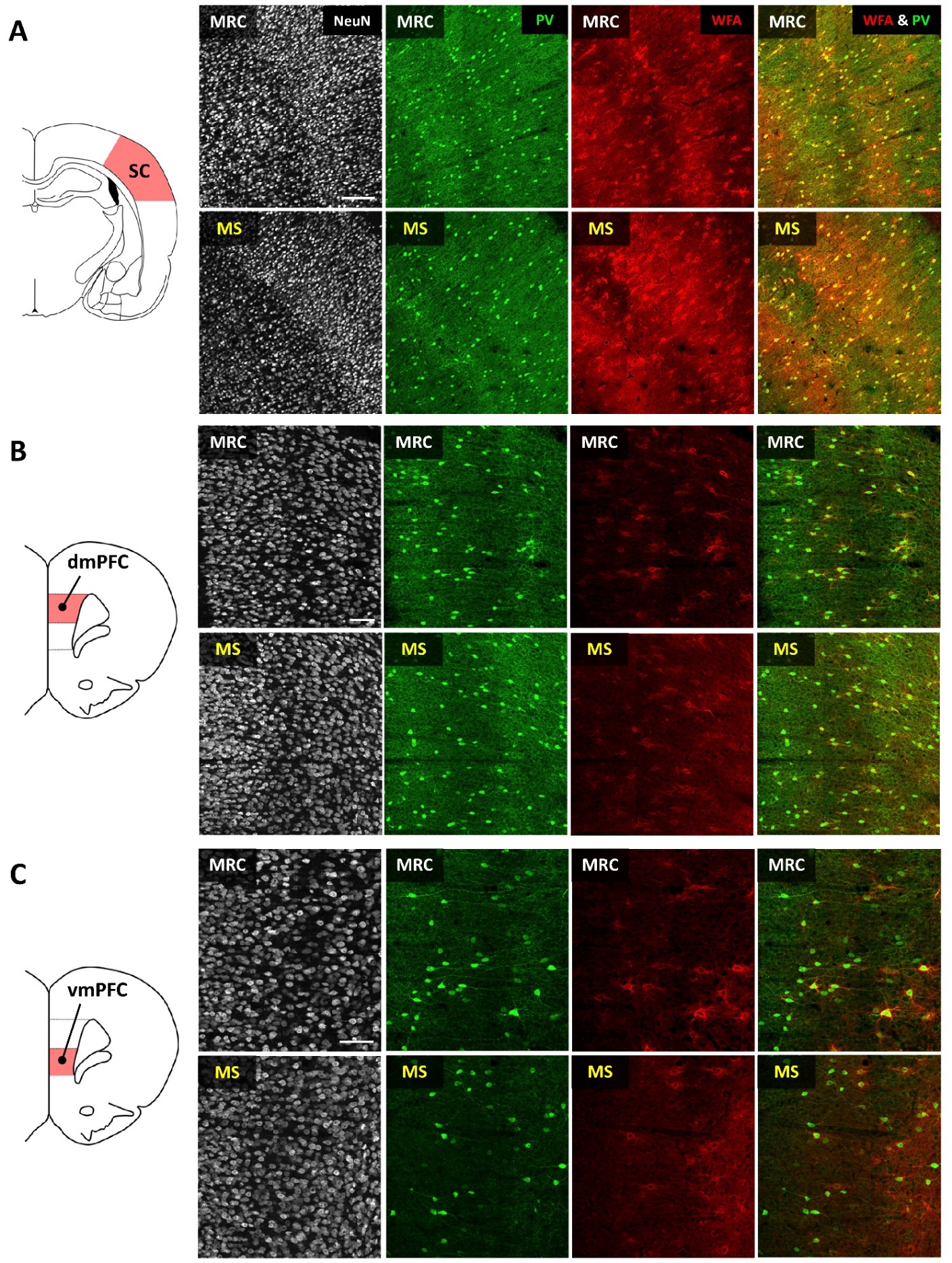
**

**Figure S2.** Low-magnification images of the sensory cortex SC; A, scale bar = 200 μm), dorsomedial prefrontal cortex (dmPFC; B, scale bar = 100 μm), and ventromedial prefrontal cortex (vmPFC; C, scale bar = 100 μm). Contrast adjustment of images (A, B, and C) was performed using the identical settings based on the SC as in Figure 6. NeuN: neuronal nuclei, PV: parvalbumin, WFA: *Wisteria floribunda* agglutinin.

# Supplemental Data 4: Immunostaining Image at Low Magnification of Figures 7 and 8

**
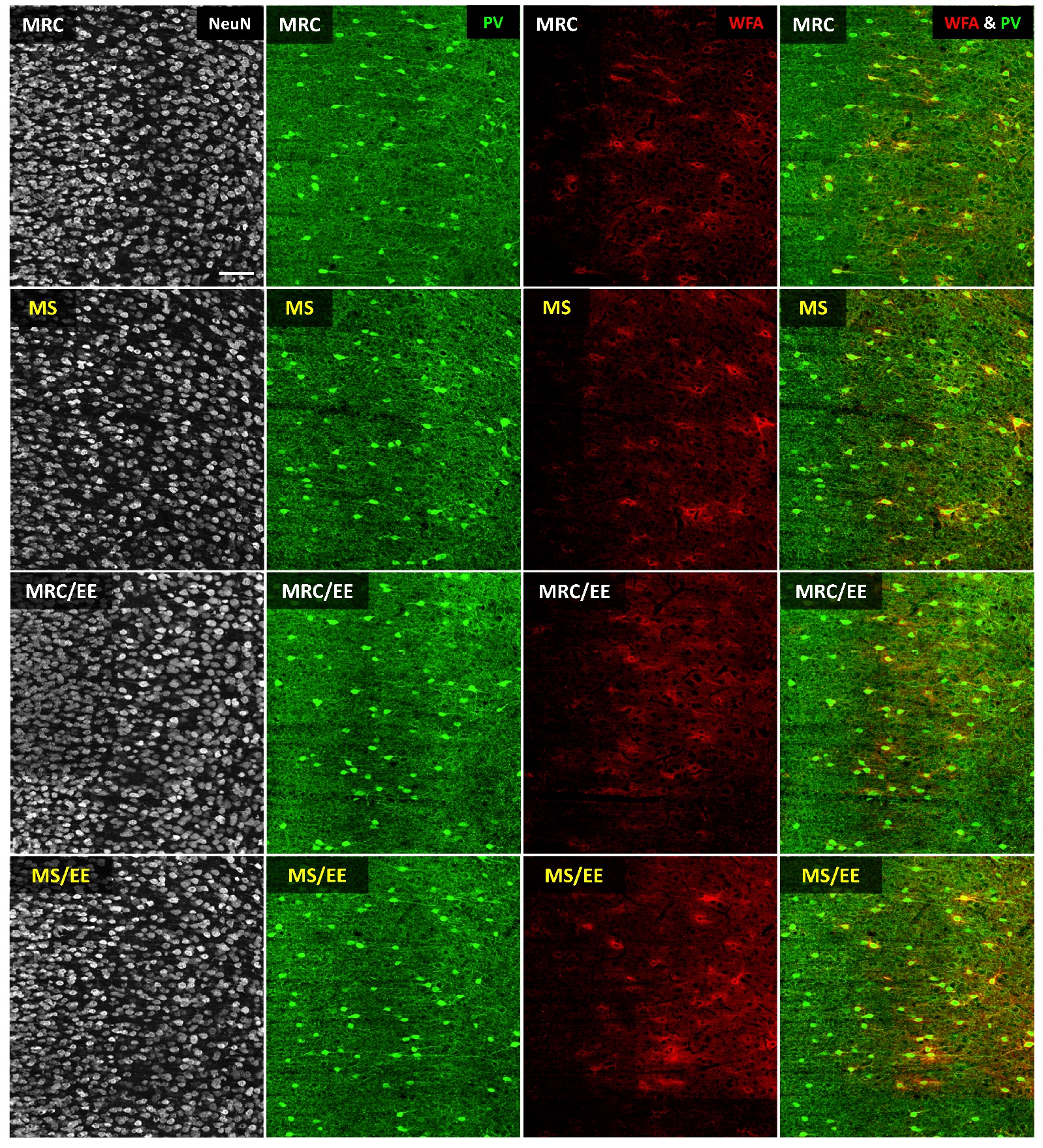
**

**Figure S3.** Low-magnification images of the dorsomedial prefrontal cortex (dmPFC) in Figure 7. Scale bar = 100 μm. Contrast adjustment of images was performed using identical settings based on the dmPFC as in Figure 7. NeuN: neuronal nuclei, PV: parvalbumin, WFA: *Wisteria floribunda* agglutinin, MRC: mother-reared control, MS: maternal separation, EE: enriched environment.

**
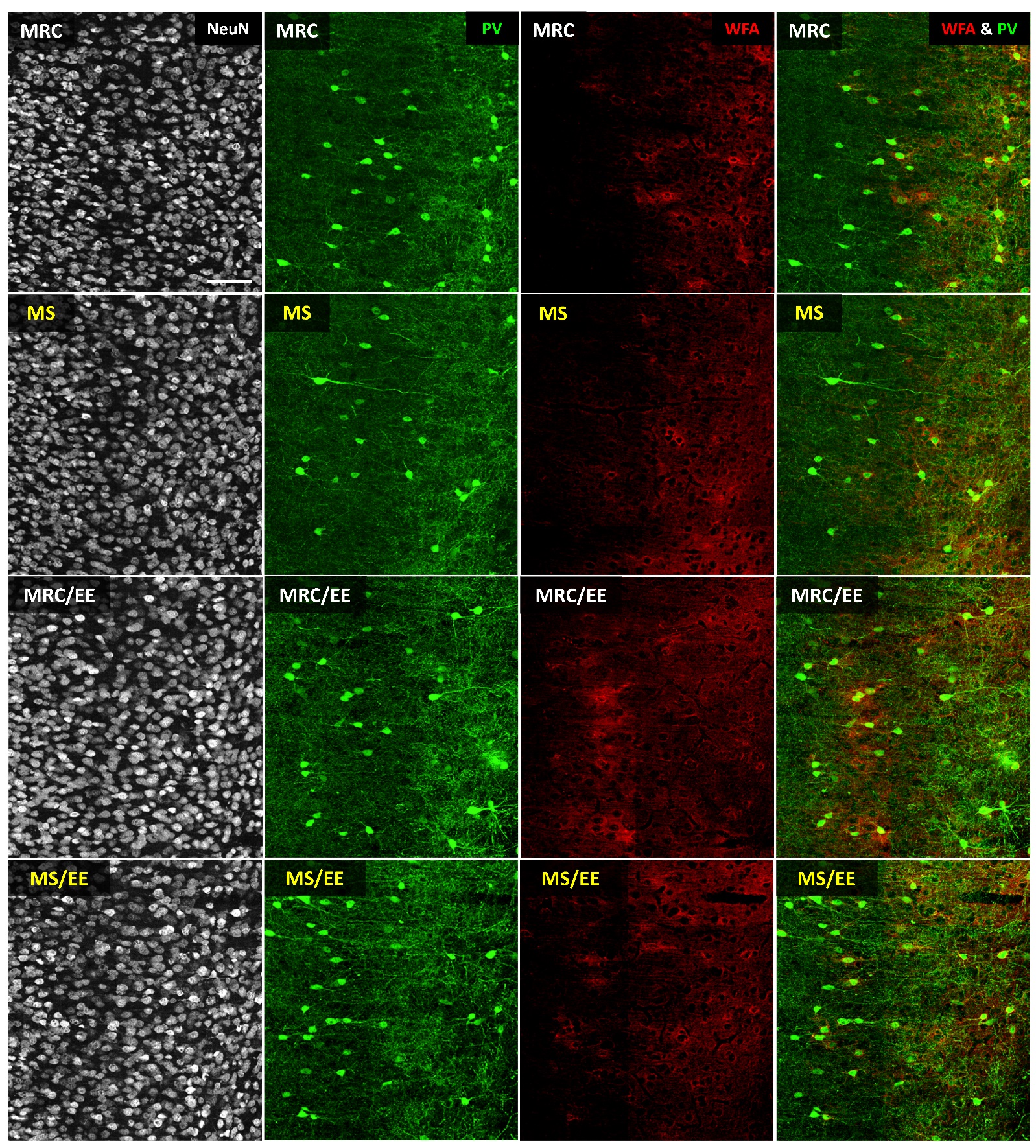
**

**Figure S4.** Low-magnification images of the ventromedial prefrontal cortex in Figure 8. Scale bar = 100 μm. Contrast adjustment of images was performed using identical settings based on the dorsomedial prefrontal cortex, as in Figure 8. NeuN: neuronal nuclei, PV: parvalbumin, WFA: *Wisteria floribunda* agglutinin, MRC: mother-reared control, MS: maternal separation, EE: enriched environment.

# Supplemental Data 5: Maternal Separation (MS) Transiently Decreases the Gene Expression Level of Brain-Derived Neurotrophic Factor (BDNF) During Early Brain Development

The procedure employed in this experiment was similar to that described in the “Materials and Methods” (sections 2.1, 2.2, 2.3, and 2.5). The primer pairs have been reported previously (Ohta *et al*., 2017; Tenkumo *et al*., 2020). The mRNA levels of *BDNF* in the MS group were lower in the sensory cortex (SC) (Fig. S5A, *z* = 3.361, *p* < 0.001, *q* = 0.001) and the medial prefrontal cortex (mPFC) (Fig. S5C, *t* (14) = 2.724, *p* = 0.016, *q* = 0.040) on PD 7 and were decreased only in the mPFC on PD 21 (Fig. S5C, *t* (14) = 3.623, *p* = 0.003, *q* = 0.015). In contrast, the mRNA level of the tropomyosin-related kinase B (*TrkB*) receptor in the MS group was decreased only on PD28 in the SC (Fig. S5B, *t* (14) = 3.415, *p* = 0.004, *q* = 0.004). All statistical results are shown in Table S10.


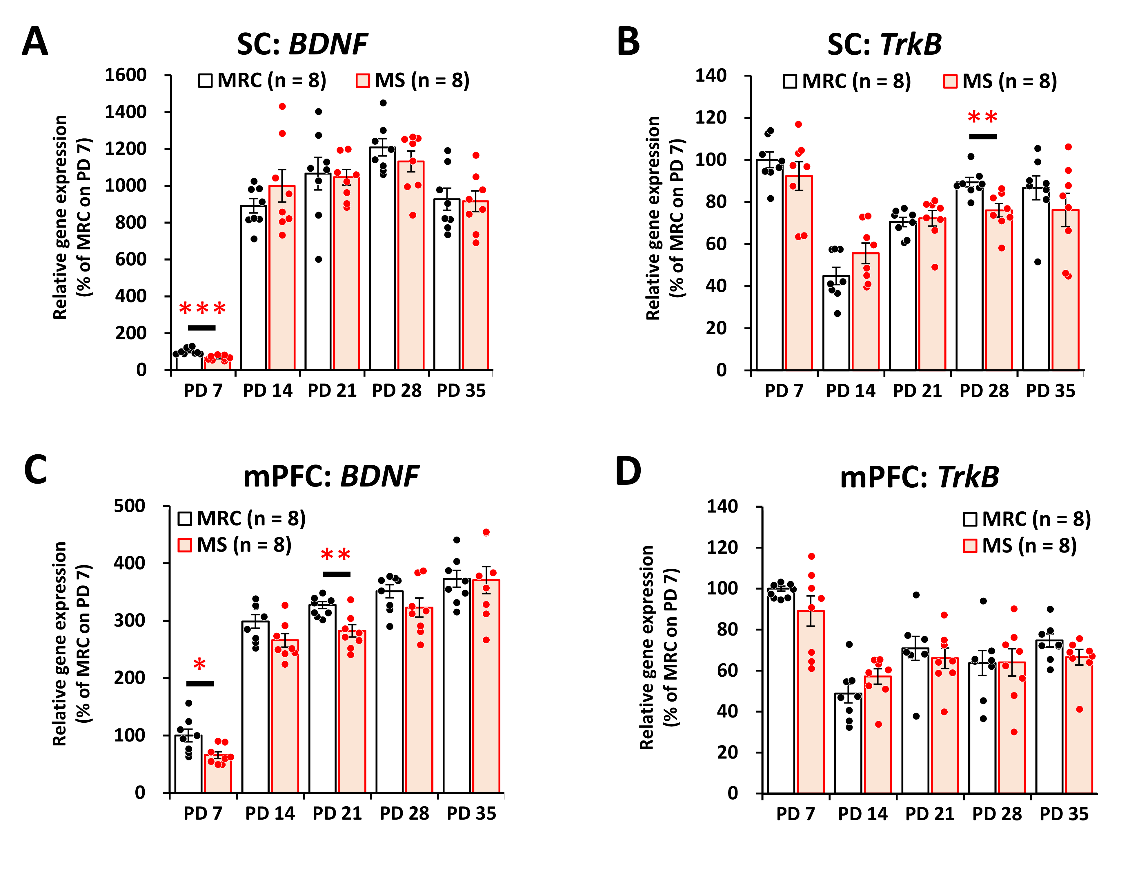


**Figure S5.** Maternal separation (MS) decreased the mRNA level of brain-derived neurotrophic factor (*BDNF*) during early brain development. (A) *BDNF* gene expression in the SC until PD 35. (B) *TrkB* gene expression in the SC until PD 35. (C) *BDNF* expression in the mPFC until PD 35. (D) *TrkB* gene expression in the mPFC. The data were obtained from eight animals per group and are expressed as mean ± SE. Student's t-test or Mann-Whitney U test was used to determine statistically significant differences between the mother-reared control (MRC) and MS groups. When a significant difference was observed in gene expression between the two groups during PD 7–35, the *q*-value was evaluated among the results between PD 7–35 using 5% FDR (*q*-value < 0.05) as the threshold. **p* < 0.05, ***p* < 0.01, ****p* < 0.001.

**Table S10.** Results of statistical analysis of data in Figure S5.

**References**

Ohta, K.I., Suzuki, S., Warita, K., Kaji, T., Kusaka, T., and Miki, T. (2017). Prolonged maternal separation attenuates BDNF-ERK signaling correlated with spine formation in the hippocampus during early brain development. *J Neurochem* 141(2)**,** 179-194. doi: 10.1111/jnc.13977.

Tenkumo, C., Ohta, K.I., Suzuki, S., Warita, K., Irie, K., Teradaya, S., et al. (2020). Repeated maternal separation causes a transient reduction in BDNF expression in the medial prefrontal cortex during early brain development, affecting inhibitory neuron development. *Heliyon* 6(8)**,** e04781. doi: 10.1016/j.heliyon.2020.e04781.

# Supplemental Data 6: Correlation between gene expressions in the medial prefrontal cortex and sensory cortex on each postnatal day

The Pearson correlation coefficient was calculated to analyze correlations between each gene expression. However, Spearman’s rank correlation coefficient was calculated as the correlation analysis when normal distribution was not confirmed via the Shapiro–Wilk test.


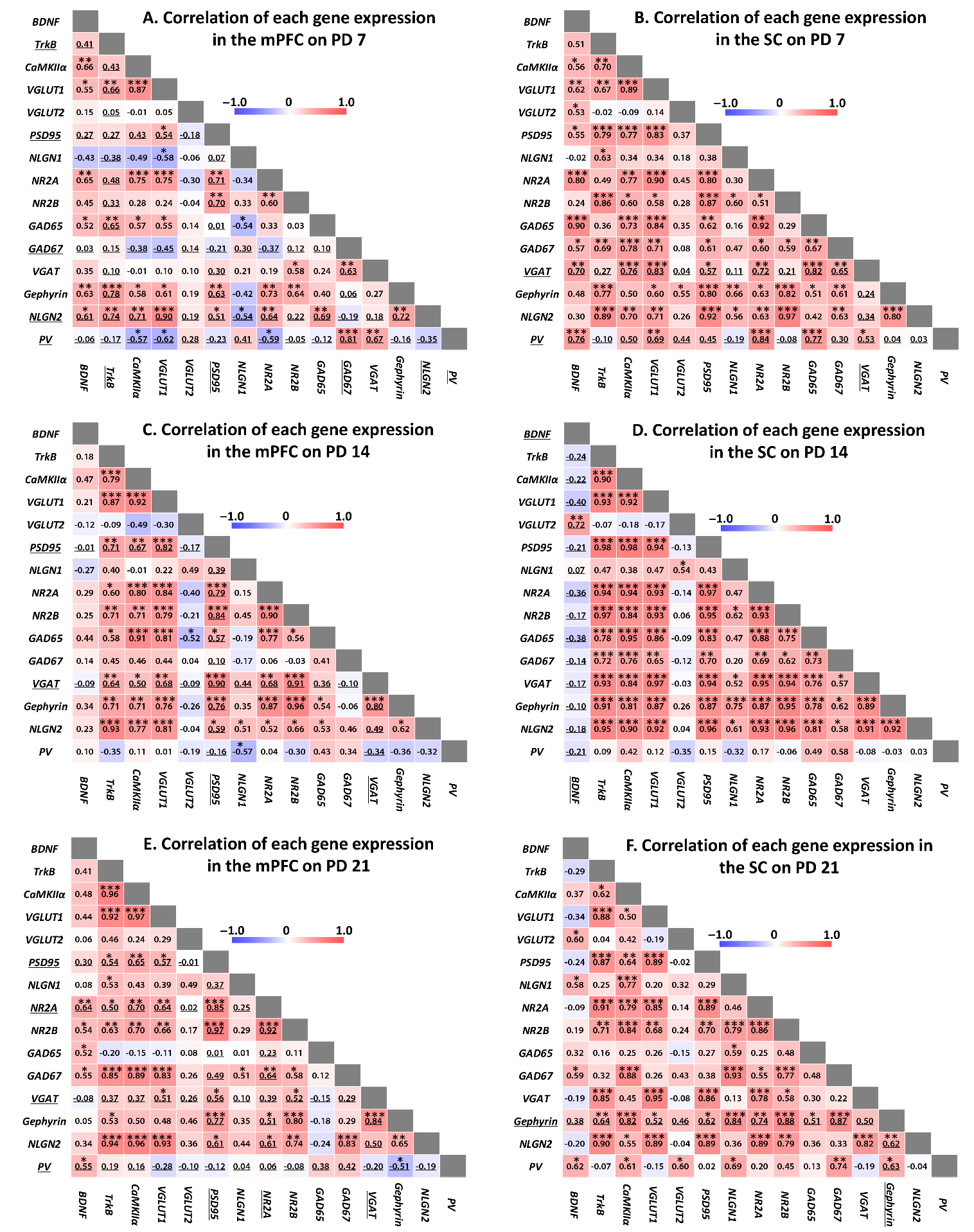


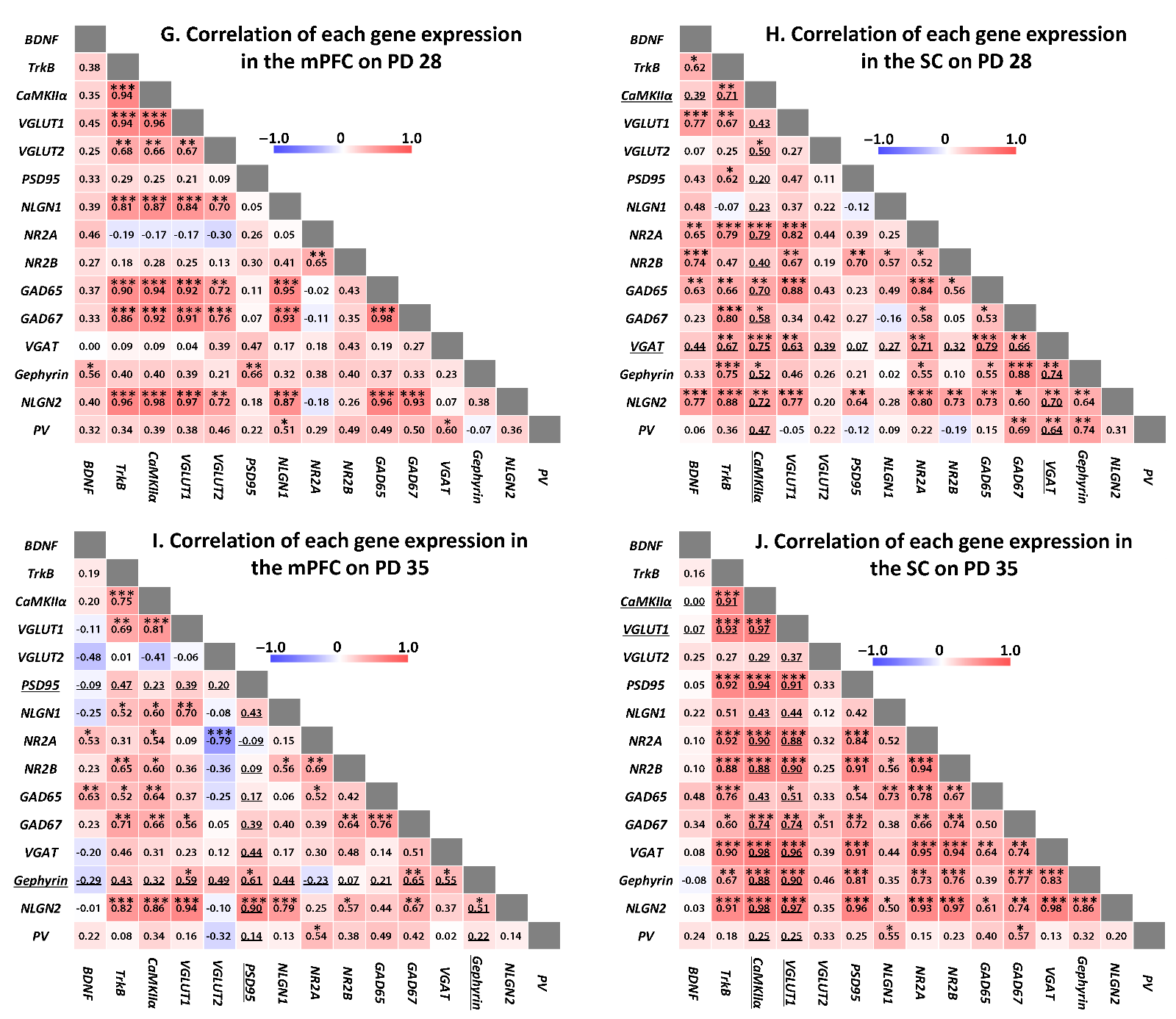


**Figure S6.** Correlation between each gene expression in the medial prefrontal cortex (mPFC) and sensory cortex (SC) during PDs 7–35. Underlined values indicate non-normally distributed data and Spearman’s rank correlation coefficient was calculated for the correlation analysis. **p* < 0.05, ***p* < 0.01, ****p* < 0.001.

# Supplemental Data 7: Maternal separation (ms) has no impact on the number of parvalbumin (PV)^+^ interneurons in the lateral/basolateral amygdala and hippocampus on PD 35

The procedure employed in this experiment was similar to that described in Sections 2.1, 2.2, 2.4, and 2.5. For the analysis of the lateral/basolateral amygdala (LA/BLA) and hippocampus (Hip), three slices were evaluated at the same bregma level as the sensory cortex (bregma −2.72, −2.88, and −3.04 mm). No significant differences were observed in the number of NeuN^+^ cells (Fig. S6A), PV^+^ cells (Fig. S7B), and the ratio of PV^+^ *Wisteria floribunda* agglutinin (WFA)^+^ to PV^+^ cells (Fig. S7C) in the LA/BLA and Hip between the mother-reared control (MRC) and MS groups. Immunostaining images are shown in Fig. S8 (LA/BLA) and Fig. S9 (Hip).

**
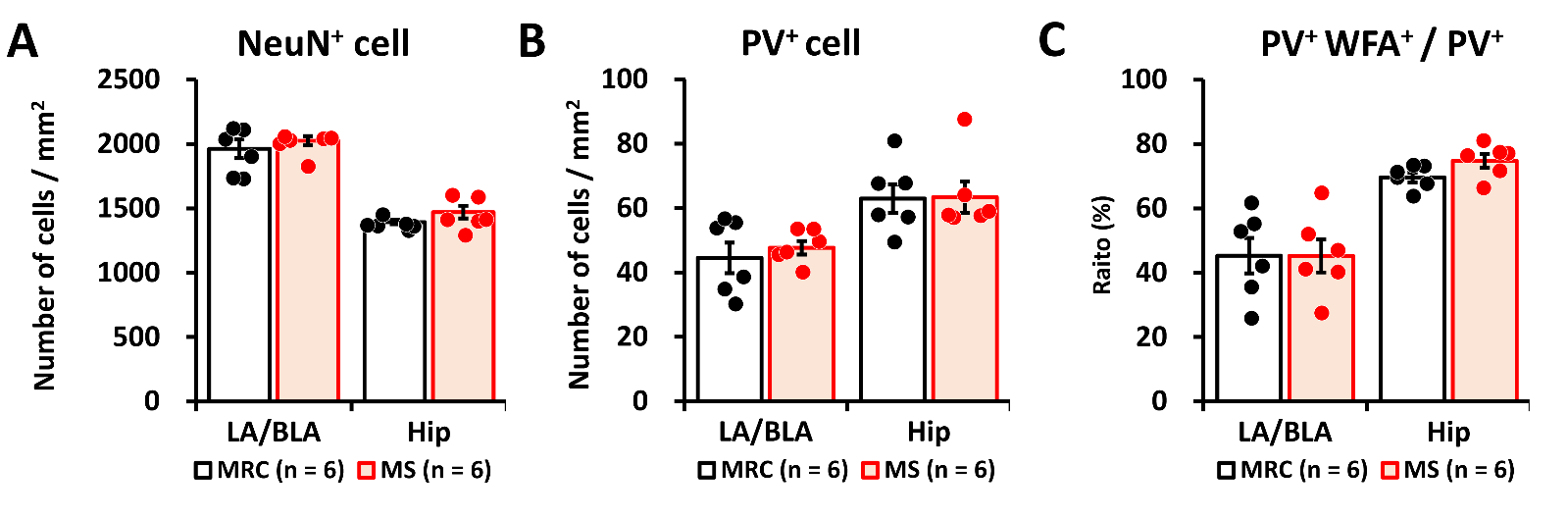
**

**Figure S7.** Maternal separation (MS) does not impact the number or maturation of parvalbumin (PV)^+^ cells in the lateral/basolateral amygdala (LA/BLA) and hippocampus (Hip) on PD 35. (A) The number of neuronal nuclei (NeuN)^+^ cells in both areas on PD 35. (B) The number of PV^+^ cells in both areas on PD 35. (C) The ratio of PV^+^ *Wisteria floribunda* agglutinin (WFA)^+^ cells /PV^+^ cells on PD 35. The data were obtained from six animals per group and are expressed as mean ± SE. Student's t-test or Mann–Whitney U test was used to determine statistically significant differences between the mother-reared control (MRC) and MS groups.


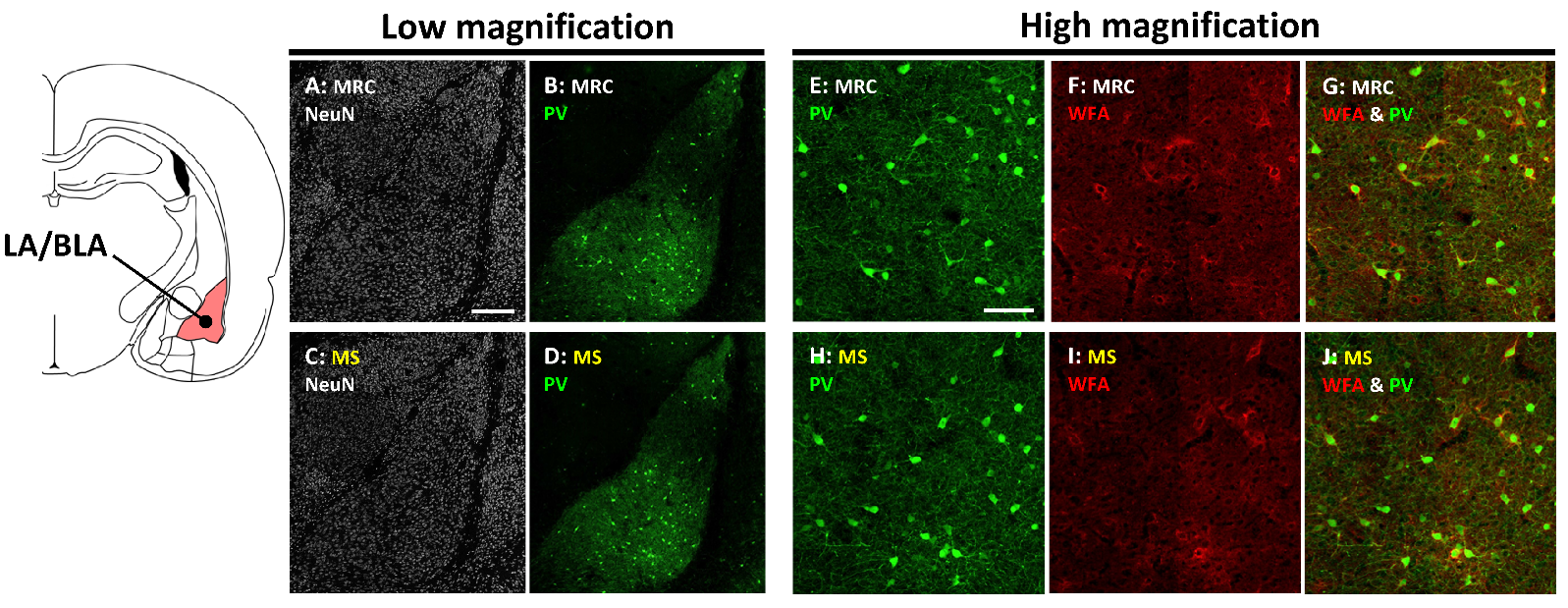


**Figure S8.** Immunostaining images of both groups in the lateral/basolateral amygdala (LA/BLA). (A–D) Immunostaining image at low magnification. Scale bar = 300 μm. (E–J) Immunostaining image at high magnification. Scale bar = 100 μm. NeuN: neuronal nuclei, PV: parvalbumin, WFA: *Wisteria floribunda* agglutinin, MRC: mother-reared control, MS: maternal separation.


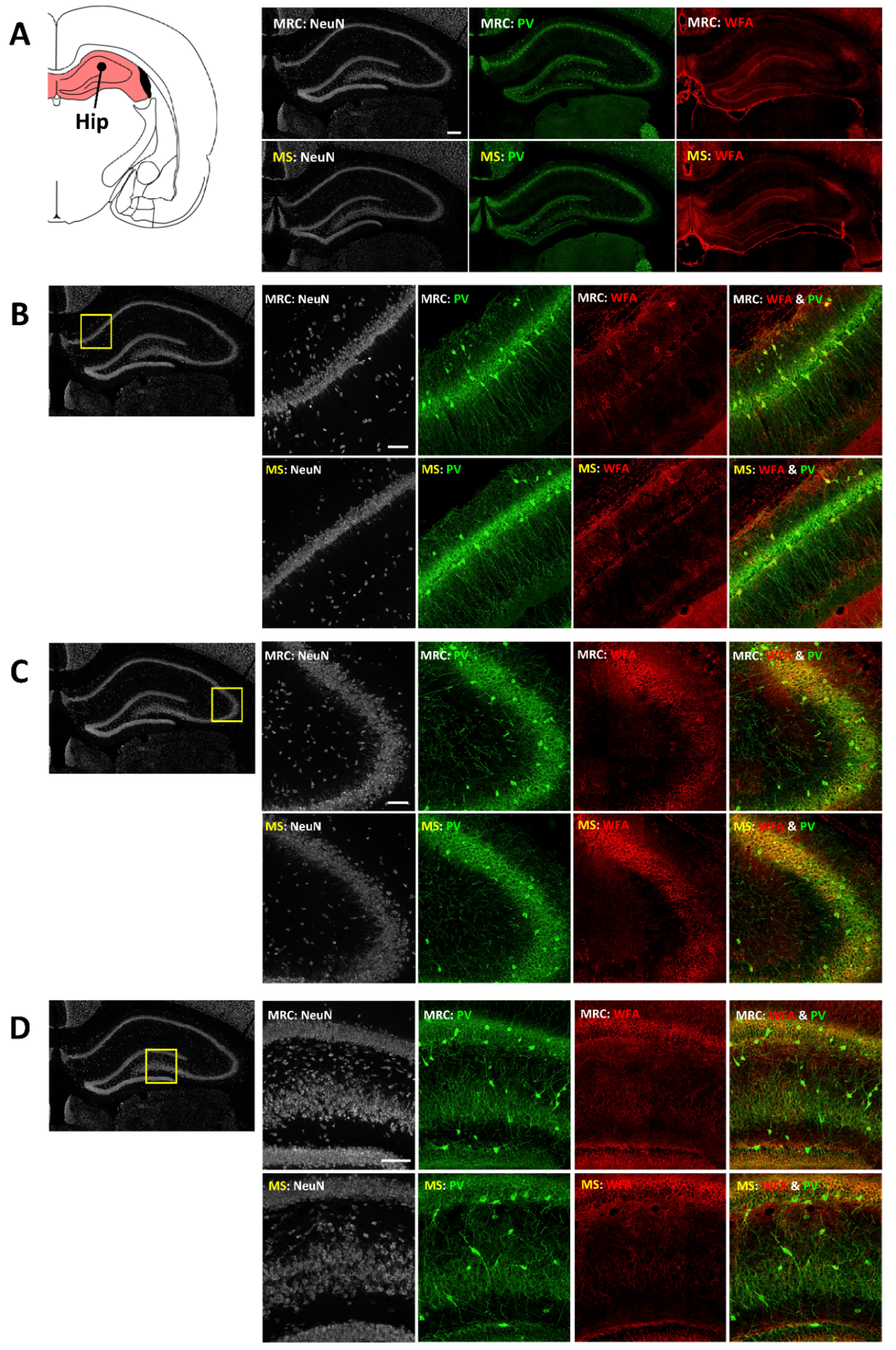


**Figure S9.** Immunostaining images of both groups in the hippocampus (Hip). (A) Immunostaining image at low magnification. Scale bar = 300 μm. (B-D) Immunostaining image at high magnification in each area. Scale bar = 100 μm. NeuN: neuronal nuclei, PV: parvalbumin, WFA: *Wisteria floribunda* agglutinin, MRC: mother-reared control, MS: maternal separation.
